# Highly Absorbing Lead-Free Semiconductor Cu_2_AgBiI_6_ for Photovoltaic Applications from the Quaternary CuI–AgI–BiI_3_ Phase Space

**DOI:** 10.1021/jacs.1c00495

**Published:** 2021-03-08

**Authors:** Harry C. Sansom, Giulia Longo, Adam D. Wright, Leonardo R. V. Buizza, Suhas Mahesh, Bernard Wenger, Marco Zanella, Mojtaba Abdi-Jalebi, Michael J. Pitcher, Matthew S. Dyer, Troy D. Manning, Richard H. Friend, Laura M. Herz, Henry J. Snaith, John B. Claridge, Matthew J. Rosseinsky

**Affiliations:** † University of Liverpool, Department of Chemistry, Crown Street, Liverpool L69 7ZD, U.K.; ‡ University of Oxford, Clarendon Laboratory, Department of Physics, Parks Road, Oxford OX1 3PU, U.K.; § University College London, Institute for Materials Discovery, Torrington Place, London WC1E 7JE, U.K.; ∥ University of Cambridge, Cavendish Laboratory, JJ Thomson Avenue, Cambridge CB3 0HE, U.K.

## Abstract

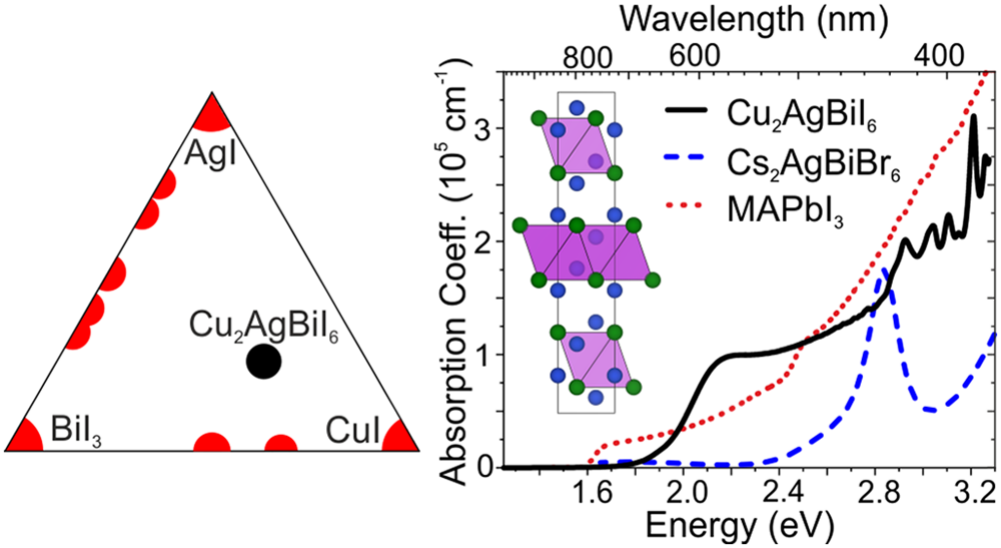

Since the emergence of lead halide perovskites for photovoltaic research, there has been mounting effort in the search for alternative compounds with improved or complementary physical, chemical, or optoelectronic properties. Here, we report the discovery of Cu_2_AgBiI_6_: a stable, inorganic, lead-free wide-band-gap semiconductor, well suited for use in lead-free tandem photovoltaics. We measure a very high absorption coefficient of 1.0 × 10^5^ cm^–1^ near the absorption onset, several times that of CH_3_NH_3_PbI_3_. Solution-processed Cu_2_AgBiI_6_ thin films show a direct band gap of 2.06(1) eV, an exciton binding energy of 25 meV, a substantial charge-carrier mobility (1.7 cm^2^ V^–1^ s^–1^), a long photoluminescence lifetime (33 ns), and a relatively small Stokes shift between absorption and emission. Crucially, we solve the structure of the first quaternary compound in the phase space among CuI, AgI and BiI_3_. The structure includes both tetrahedral and octahedral species which are open to compositional tuning and chemical substitution to further enhance properties. Since the proposed double-perovskite Cs_2_AgBiI_6_ thin films have not been synthesized to date, Cu_2_AgBiI_6_ is a valuable example of a stable Ag^+^/Bi^3+^ octahedral motif in a close-packed iodide sublattice that is accessed via the enhanced chemical diversity of the quaternary phase space.

## 1 Introduction

Hybrid lead perovskites APb^2+^X_3_ (A = FA^+^, MA^+^, Cs^+^; X = Br^–^, I^–^) continue to be intensely studied as solar absorbers for photovoltaic (PV) applications due to their high absorption coefficients suitable for thin-film technology,^1−3^ long charge-carrier diffusion lengths,^4−7^ and high radiative efficiencies. In single-junction devices the current certified record power conversion efficiency (PCE) stands at 25.5%.^8^ This is close to matching the very highest efficiencies delivered by silicon PV cells, and high PCEs are a way to minimize the cost of energy from PVs. However, this becomes increasingly difficult, as heavily optimized systems approach their maximum theoretical efficiency limits. A crucial strategy to overcome this limitation is to combine suitably wide band gap (*E*_g_) materials (*E*_g_ from 1.6 to 2.0 eV) with well-established c-Si (*E*_g_ ≈ 1.1 eV) technology to construct tandem cells, which can achieve much higher PCEs in comparison to single-junction cells.^9,10^

The tunability of the band gap in mixed iodide–bromide lead halide perovskites has opened up the possibility of multijunction solar cells with c-Si, currently delivering a record PCE of 29.5%, with efficiency improvements to over 32% being feasible.^9,11^ There remain a number of compromises which could be improved upon with the discovery of new wide-band-gap, stable, lead-free, inorganic solar absorber materials. These include the yet unresolved challenge of obtaining band-gap-stable, low-defect I–Br mixed halide perovskites,^12^ the reliance upon organic ammonium cations to deliver a crystallographically phase stable lead halide perovskite compound, which leads to lower thermal stability in comparison to conventional inorganic semiconductors, and finally the fact that these materials contain lead, which requires careful management due to the known toxicological issues. One strategy for replacing Pb^2+^ is with isoelectronic Bi^3+^. Bismuth bromide and chloride networks have been synthesized as the double perovskites (MA)_2_KBiCl_6_ (3.04 eV),^13^ (MA)_2_AgBiBr_6_ (2.02 eV),^14^ Cs_2_AgBiCl_6_ (2.77 eV), and Cs_2_AgBiBr_6_ (2.19 eV);^15,16^ however, their absorption profiles remain unsuitable for use in tandem cells. Bismuth iodides A_3_Bi_2_^3+^I_9_ (A = K^+^, Rb^+^, Cs^+^, MA^+^, NH_4_^+^)^17−22^ have been reported as 2D perovskites (A = NH_4_^+^, K^+^, Rb^+^) or as 0D isolated [Bi_2_I_9_]^3+^ units (A = Cs^+^, MA^+^), which are not ideal for isotropic charge transport and carrier mobility. Hypothetical bismuth iodide double perovskites, such as Cs_2_AgBiI_6_, would possess a lower, more ideal band gap but so far have not been stable enough to be synthesized,^23^ apart from in nanocrystal form,^24^ which identifies a clear opportunity for materials discovery. Searching for other possible bismuth iodide networks with suitably lower band gaps leads to BiI_3_ and the ternary compounds Ag_1–3*x*_Bi_1+*x*_I_4_ and CuBiI_4_. BiI_3_ has been reported with an indirect band gap of 1.67(1) eV,^25^ and PV devices have reached PCEs of 1.0%.^26−28^ Ag_1–3*x*_Bi_1+*x*_I_4_ and CuBiI_4_ have been reported with suitable band gaps of 1.64–1.93 eV; the variation arises from composition, sample type, and assumption of direct or indirect band gaps.^25,29,30^ Devices based on a *x* = −0.33 Ag_1–3*x*_Bi_1+*x*_I_4_(Ag_3_BiI_6_) solar absorber have reached PCEs of 4.3%,^31^ and introducing small amounts of sulfur to the layer has recently been shown to increase the *J*_sc_ values of devices, increasing the maximum PCE to 5.44(7)%.^32^ Cu-containing CuBiI_4_ films have also recently been processed into devices reaching PCEs of 1.1%.^30,33^ However, we show here that CuBiI_4_ is not a stable phase and decomposes on standing at room temperature. As with the initial reports of MAPbI_3_ devices, the low initial PCEs of devices using these recent materials are a result of limited investigations into optimal device architectures, charge carrier layers, control of crystallinity and passivation, and further materials chemistry. Here, we synthesize the new compound Cu_2_AgBiI_6_ as crystals, powders, and solution-processed thin films, solve its crystal structure, and present its properties. By expanding this family of materials to the quaternary Cu–Ag–Bi–I system, we gain an extra degree of chemical tunability, which can be further optimized to increase the performance and stability of this lead-free absorber material. Cu_2_AgBiI_6_ represents the use of Ag^+^ to stabilize CuBiI_4_ and the use of Cu^+^ to reduce the content of Ag^+^ in comparison to Ag_1–3*x*_Bi_1+*x*_I_4_ compounds.

## 2 Results

### 2.1 Cu_2_AgBiI_6_ Crystal Structure

**Figure 1:**
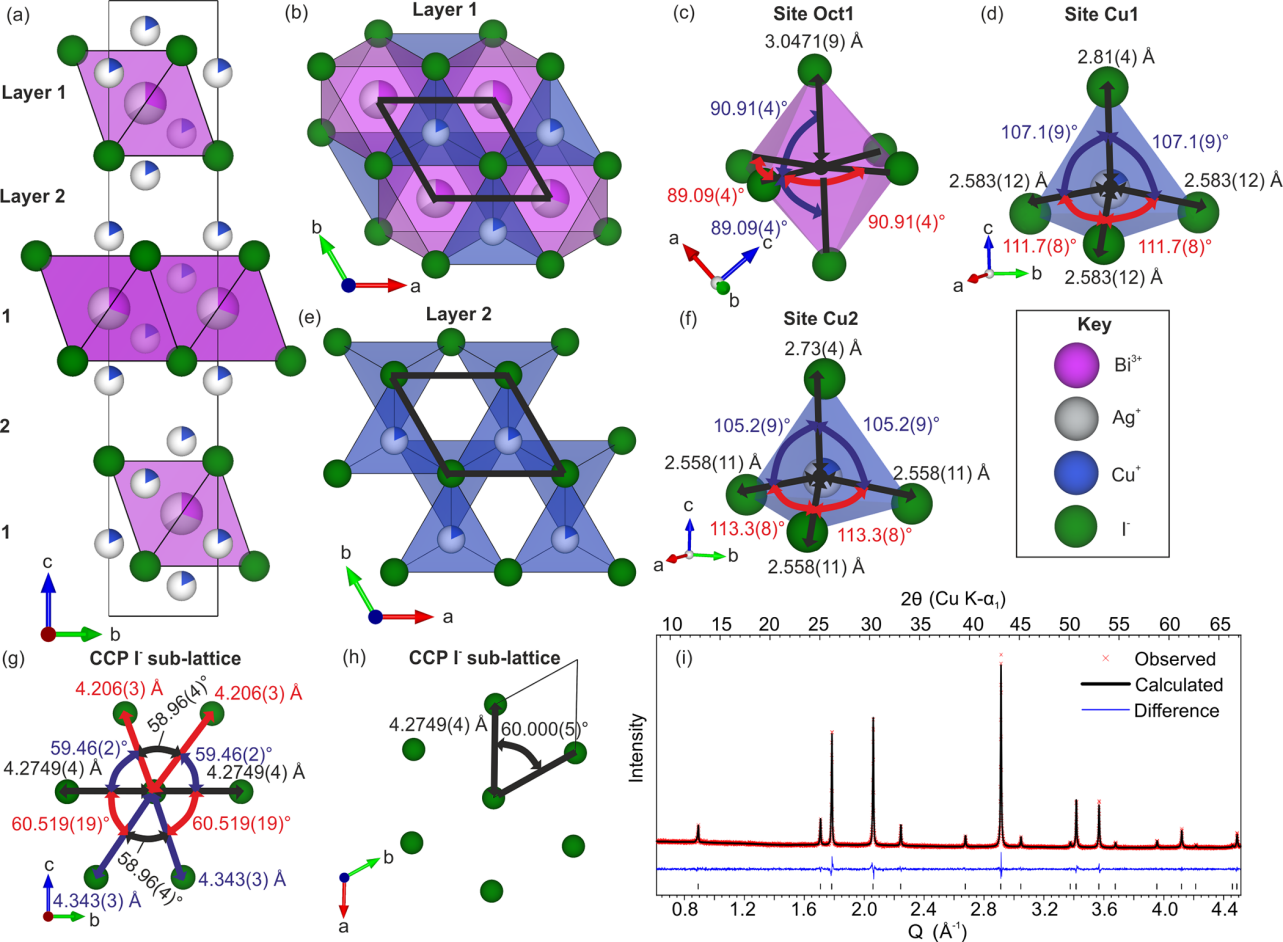
(a) Structure of Cu_2_AgBiI_6_ solved from 100 K SCXRD data with the composition constrained in line with the average composition Cu_2.15(16)_Ag_1.04(5)_Bi_0.92(7)_I_6.00(11)_ from TEM EDX. (b) Layer 1 contains the sites Oct1 (occupied by 34.6% Ag^+^ and 30.6% Bi^3+^) and Cu1 with coordination environments shown in (c) and (d), respectively. (e) Layer 2 contains the site Cu2 with the coordination environment shown in (f). Sites Cu1 and Cu2 are both occupied by 17.9% Cu^+^. The I–I distances and I–I–I angles of the cubic close-packed (CCP) iodide sublattice in the *bc* and *ab* planes are shown in (g) and (h), respectively. The green, blue, gray, and pink spheres/polyhedra represent I^–^, Cu^+^, Ag^+^ and Bi^3+^ ions, respectively. (i) Pawley fit performed on room-temperature laboratory PXRD data.

**Figure 2:**
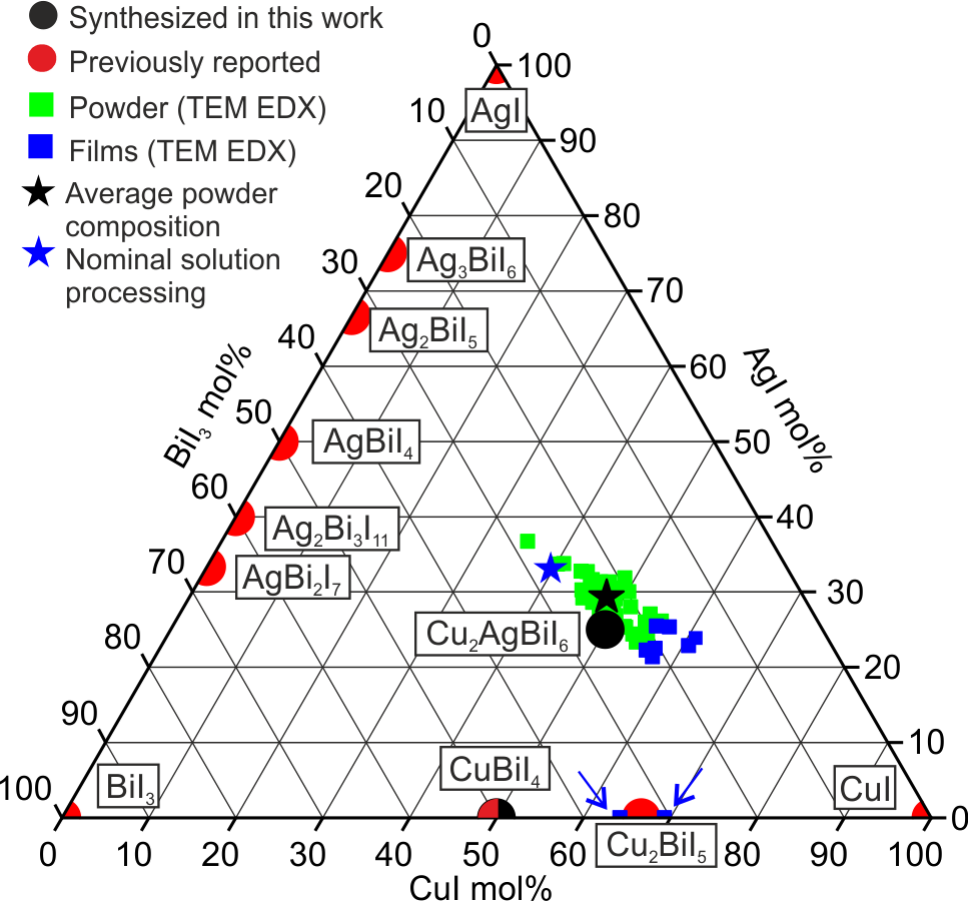
Compounds of the CuI–AgI–BiI_3_ phase space. The previously reported compounds are shown in red, including Ag_1–3*x*_Bi_1+*x*_I_4_, CuBiI_4_, and Cu_2_BiI_5_.^34−38^ Shown in black are the phases synthesized here, including Cu_2_AgBiI_6_, the first report of a quaternary phase in the CuI–AgI–BiI_3_ phase space. The TEM EDX measurements of powder samples shown in green give an average composition of Cu_2.15(16)_Ag_1.04(5)_Bi_0.92(7)_I_6.00(11)_ (black star). The composition dissolved in solution for processing thin films is shown by a blue star. The TEM EDX measurements of films are shown in blue, with a Cu_2_AgBiI_6_ main phase, and an impurity phase of Cu_2_BiI_5_, indicated by the blue arrows.

We synthesized Cu_2_AgBiI_6_ powders and crystals by a solid-state synthesis in evacuated fused-silica ampules as described in the Supporting Information. We found that it is important to quench the material from the synthesis temperature of 350 °C rather than cool it down slowly through a range of temperatures, which induces compositional heterogeneity as measured by the TEM EDX (Figure S1). Crystals were picked from the powder reaction and were found to be of suitable quality for single-crystal X-ray diffraction (SCXRD) (Figure S2). Larger crystals grown by chemical vapor transport and cooling of the melt were found to contain large amounts of heterogeneity and twinning. We solved the structure of Cu_2_AgBiI_6_ using SCXRD data collected at 100 K (Figure 1, Table 1 and Tables S2 and S3). The observed reflections could be fitted with a twinning of four trigonal unit cells with space group *R*3*m* and lattice parameters *a* = 4.2749(3) Å and *c* = 20.9395(16) Å, which is metrically cubic within 2σ error (). The trigonal unit cell and definition of rhombohedral strain are shown in Figure S3a,b. Table S1 gives the contribution of each twin and the twinning matrices. The twinning is complex and has been reported in more detail for AgBiI_4_,^25^ which, due to the twinning, has two indistinguishable structural solutions—a defect spinel and/or twinning of a CdCl_2_ structure. Here, we find that Cu_2_AgBiI_6_ consists of a cubic close-packed (CCP) iodide sublattice (Figure S3a), as reported for the Ag_1–3*x*_Bi_1+*x*_I_4_ and CuBiI_4_ materials. The octahedral cations Ag^+^ and Bi^3+^ then adopt a CdCl_2_ octahedral motif in a disordered fashion (Figure S3c). This consists of layers of 2D edge-sharing octahedra separated by a layer of vacant octahedral sites. The atomic occupancies of Ag^+^ and Bi^3+^ are 34.7% and 30.6%, respectively. Rather than a direct refinement of the composition, the Ag^+^ and Bi^3+^ occupancies were constrained to the average composition Cu_2.15(16)_Ag_1.04(5)_Bi_0.92(7)_I_6.00(11)_—the composition of the powder measured by TEM EDX (Figure 2). This compositional constraint was required due to the high number of correlated parameters in the refinement derived from cation disorder and the four twin components. A comparison to AgBiI_4_ shows that adding tetrahedral Cu^+^ in to the structure has reduced the octahedral occupancy of the Ag^+^ and Bi^3+^ and introduced octahedral vacancies to maintain charge balance. The formula Cu_4*x*_(AgBi)_1–*x*_I_4_ expresses this case, where equal amounts of Ag^+^ and Bi^3+^ are substituted for Cu^+^, with *x* = 0.33 corresponding to Cu_2_AgBiI_6_. The electron density in the difference Fourier map shows two Cu^+^ sites (Cu1 and Cu2) with equal occupancy (Figure S3d). They occupy every possible tetrahedral site in the CCP iodide sublattice, as in the reported CuBiI_4_ structure.^35^ The Cu^+^ atomic occupancies were fixed to occupancies of 17.9%, in line with the measured composition. Cu_2_AgBiI_6_ provides the initial report and understanding of a quaternary phase in the CuI–AgI–BiI_3_ phase space (Figure 2). The Cu_2_AgBiI_6_ structure is analogous with oxides, where occupancy of this pattern of tetrahedral sites within the *R*3*m* space group and CdCl_2_-type octahedral site occupancy motif have been observed: for example, in nonstoichiometric lithium vanadium oxides such as Li_0.22_VO_2_, which have cationic disorder due to delithiation.^39^ We performed a Pawley fit on room-temperature laboratory powder X-ray diffraction (PXRD) data of Cu_2_AgBiI_6_, yielding lattice parameters of *a* = 4.3151(2) Å and *c* = 21.141(1) Å (Figure 1i), which is metrically cubic within error ().

**Table 1:** Refined Structural Data for the Cu_2_AgBiI_6_ Structure Solved from 100 K SCXRD Data with the Composition Constrained to Match the Average Composition Cu_2.15(16)_Ag_1.04(5)_Bi_0.92(7)_I_6.00(11)_ from TEM EDX^a^

site	atom	*x*	*y*	*z*	occ	*U* (10^3^ Å^2^)	Wyckoff position	point group (Hermann– Mauguin)
I1	I	2/3	1/3	0.08133(7)	1	16.6(7)	6c	3*m*
Oct1	Bi	1/3	2/3	1/6	0.306	24.0(9)	3b	3̅*m*
						24.0(9)		
	Ag	1/3	2/3	1/6	0.347		3b	3̅*m*
Cu1	Cu	0	0	0 1177(19)	0.179	22	6c	3*m*
Cu2	Cu	2/3	1/3	–0.0492(19)	0.179	22	6c	3m

a Crystal data: Cu_2_AgBilI_6_, space group *R*3̅*m* (No. 166), 100 K, formula sum Cu_2.15_Ag_1.04_Bi_0.92_I_6_, *Z* = 1, formula mass 1202.46 g/mol, cell parameters *a* = 4.2749(3) Å and *c* = 20.9395(16) Å, trigonal crystal system, cell volume 331.40(5) Å^3^, calculated density 6.025 g/cm^3^.

### 2.2 Bulk Stability

Powders of the previously reported CuBiI_4_ and AgBiI_4_ were obtained by a solid-state synthesis in evacuated fused-silica ampules as described in the Supporting Information. The PXRD pattern of CuBiI_4_ was fitted to a cubic unit cell with the lattice parameter *a* = 12.1580(2) Å, larger than the *a* = 12.134(6) Å reported by Fourcroy et al. (Figure S4).^35^ SEM EDX confirmed a composition of Cu_1.21(5)_Bi_1.11(7)_I_4.00(9)_, within 3σ error of CuBiI_4_ (Figure S5). We find that CuBiI_4_ is a metastable material that decomposes back to the starting materials BiI_3_ and CuI at room temperature, even in the dark (Figure S6). We could slow the rate of decomposition of CuBiI_4_ by storing the powder at −20 °C. In contrast, we find that Cu_2_AgBiI_6_ powder is stable when it is kept in the dark, in air, at room temperature. We exposed synthesized Cu_2_AgBiI_6_ and AgBiI_4_ powders to a simulated AM1.5 solar spectrum for 1 week, sealed in capillaries with synthetic (dry) air, laboratory air, and He atmospheres. AgBiI_4_ and Cu_2_AgBiI_6_ showed no color change after 1 week in the solar spectrum and showed no signs of decomposition by PXRD (Figure S7) or Raman spectroscopy (Figure S8). The Cu_2_AgBiI_6_ composition therefore represents the stabilization of a Cu-containing bismuth iodide solar absorber and is as stable as AgBiI_4_ under the investigated conditions. This is promising, since unencapsulated devices using AgBiI_4_ absorber layers have been shown to retain 96% of their initial PCE after 1000 h of storage in air at 26% relative humidity.^43^

### 2.3 Optical Properties

We solution-processed Cu_2_AgBiI_6_ into thin films for optical property measurements and device fabrication, as we describe in the Supporting Information. The films were found to be consistently Cu rich in comparison to the nominal composition in solution, showing loss of Ag and Bi during the film processing. Therefore, the nominal Cu-poor composition in solution reported here (Cu_1.53_Ag_1.26_Bi_1.07_I_6.00_) was to compensate for this, bringing the compositions of films close to the composition of the powders (Figure 2). Two phases were detected in the films. The most abundant phase had a measured composition of Cu_2.52(9)_Ag_1.02(7)_Bi_0.82(11)_I_6.00(20)_. The minor phase was identified as Cu_2_BiI_5_, containing no silver (Figure 2). We performed a Pawley fit of PXRD data collected on the film (Figure S9), which shows the major phase to have a trigonal unit cell (*R*3*m*) with lattice parameters of *a* = 4.3476(8) Å and *c* = 20.868(9) Å and the impurity phase to have a trigonal unit cell (*R*3*m*), with lattice parameters *a* = 4.322(1) Å and *c* = 20.80(1) Å. This is consistent with the two phases identified in the TEM EDX. We found that the film deposition was very sensitive to the annealing temperature, which we optimized to a two-step anneal to improve the film morphology from large rough dendritic crystallites (Figure S10) to a more uniform smooth film (Figure S11).

**Figure 3:**
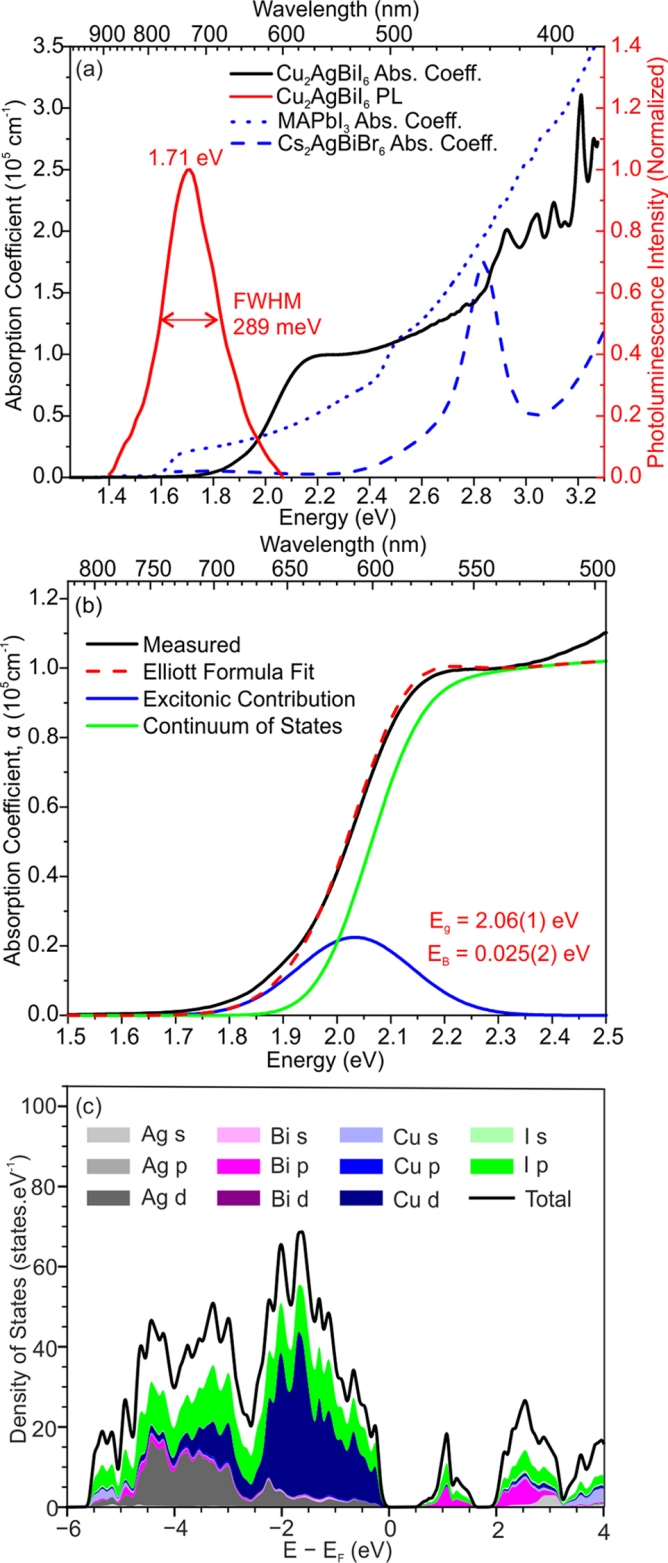
(a) Absorption coefficient of Cu_2_AgBiI_6_ thin films (black) measured by a combination of Fourier transform infrared (FTIR) spectroscopy and photothermal deflection spectroscopy (PDS). This is compared to the reported absorption coefficients of MAPbI_3_ (blue dotted line) and Cs_2_AgBiBr_6_ (blue dashed line), reproduced from Davies et al. and Longo et al., respectively.^40−42^ Also shown is the photoluminescence (PL) spectrum of Cu_2_AgBiI_6_. (b) Elliott model fitting of the absorption coefficient spectrum, giving a band gap of 2.06(1) eV and an exciton binding energy of 25(2) meV. (c) Partial density of states of Cu_2_AgBiI_6_ computed with density functional theory for configurations of cations with the lowest computed energy. The cumulative contributions from each species are shown along with the total density of states for energies relative to the computed Fermi energy.

We determined the absorption coefficient spectra of Cu_2_AgBiI_6_ thin films by using a combination of a Fourier transform infrared (FTIR) spectrometer to accurately determine the band gap absorption spectra and photothermal deflection spectroscopy (PDS) to accurately measure the low-energy parts of the spectrum. The raw PDS data (Figure S12a) were scaled to match the FTIR data, and then the two data sets were combined as shown in Figure S12b. Unusually, for this broad family of compounds, Cu_2_AgBiI_6_ presents a strong absorption coefficient profile with a steep increase typical of a direct band gap semiconductor (Figure 3a). The absorption strength that we measure for Cu_2_AgBiI_6_ at the first peak just above the band edge (1.0 × 10^5^ cm^–1^) is considerably stronger than that for MAPbI_3_ films measured here (0.3 × 10^5^ cm^–1^) and reported in the literature,^44^ which are already very strongly absorbing semiconductors near the band edge. Crucially, Cu_2_AgBiI_6_ has a much more suitable absorption profile in comparison to that of the alternative wide-band-gap, lead-free double perovskite Cs_2_AgBiBr_6_, which consists of an initial peak in the absorption spectrum centered at 2.8 eV, followed by a minimum. In the literature, it is unresolved whether this absorption peak in Cs_2_AgBiBr_6_ can be attributed to excitonic contributions or to the nature of the density of states near the band edge.^15,45−47^ Although a Tauc analysis is often used to approximate the band gap of lead halide perovskites, this is unphysical since a Tauc analysis assumes that the absorption at the band edge is directly into the continuum of states and neglects the exciton contribution that can dominate features near the band edge. The more accurate approach is a fit according to Elliott theory,^41,48^ which accounts for contributions from the excitonic contribution and continuum of states. In Figure 3b, we show a fit to the absorption coefficient based on the Elliott model, which reveals a band gap of 2.06(1) eV and an exciton binding energy (*E*_B_) of 25(2) meV. This value of the exciton binding energy is higher than that determined for MAPbI_3_ but very similar to that determined for CsPbBr_3_ and notably comparable to the thermal energy at room temperature.^49,50^ This indicates that under light absorption at room temperature free carriers, as opposed to bound excitons, will be generated. Therefore, we have stabilized a close-packed iodide framework with three metal species that together give high absorption, and both the band gap and exciton binding energies are lower than those in a comparable bromide (Cs_2_AgBiBr_6_, Tauc plot *E*_g_ ≈ 2.2 eV, *E*_B_ ≈ 220 meV).^40,51^ Both the strong absorption properties and low exciton binding energy are very encouraging for the potential use of Cu_2_AgBiI_6_ as a solar absorber, in comparison to the previously reported double perovskites. We note that, although the band gap of the continuum of states at 2.06 eV appears to be quite large for PV applications, there exists considerable absorption at lower energies due to the excitonic states. This indicates that the optical, or PV, band gap will be at lower energy.^52^ We will return to this point later on.

To gain some insight into the nature of the electronic transitions underlying optical absorption, we have performed density functional theory calculations on ordered structural models of Cu_2_AgBiI_6_ based on the refined experimental disordered structures. Partial density of states plots (Figure 3c and Figure S13) show that the bottom of the conduction band is dominated by Bi 6p and I 5p states, similar to the case for AgBiI_4_ and BiI_3_.^25^ In contrast, Cu 3d states dominate at the top of the valence band in Cu_2_AgBiI_6_, mixed with the I 5p states which dominate when Cu is absent. Optical transitions near the band gap energy of Cu_2_AgBiI_6_ will involve considerable Cu 3d to Bi 6p/I 5p character, in contrast to the I 5p to Bi 6p/I 5p transitions present in Ag_1–3*x*_Bi_1+*x*_I_4_ and BiI_3_. This suggests that the Cu^+^, which is well dispersed throughout the structure, is a functional part of the electronic network. Band structure plots for the lowest energy computed structure of Cu_2_AgBiI_6_ (Figure S21) are shown in Figure S14 and naturally reflect the precise ordering selected in the supercell used for the calculations. The in-plane effective masses of the holes and electrons are relatively low at 1.0 and 0.6 m_e_, respectively, and are similar to those calculated for AgBiI_4_.^25^ We find that the layered nature of the structure leads to flat bands in the **k**_*z*_ direction (**c** direction) in the ordered supercell studied.

In Figure 3a we also show the photoluminescence (PL) of the Cu_2_AgBiI_6_ thin film, which we fit to a pseudo-Voigt function (convolution of a Gaussian and Lorentzian function) with a full width half-maximum (fwhm) of 289 meV. The PL peak of Cu_2_AgBiI_6_ is centered at 1.71 eV, corresponding to a Stokes shift of 350 meV in comparison to the estimated direct band gap. For comparison, we show the absorption and emission profiles of MAPbI_3_ in Figure S15. We can fit the PL of MAPbI_3_ to a Gaussian function with a fwhm of 96 meV and a Stokes shift of 10 meV. Although the Stokes shift for Cu_2_AgBiI_6_ is larger than in MAPbI_3_, it is still substantially less than the 1 eV separation between the direct gap energy and PL peak in the indirect band gap material Cs_2_AgBiBr_6_.^45^ Due to the disordered nature of the Cu_2_AgBiI_6_ crystal structure, the sub-band-gap states of the thin film were investigated using PDS. PDS is a scatter-free absorption measurement capable of assessing the presence of sub-band-gap states and/or a broad distribution of states near the band edge. Interestingly, the PDS measurement reveals absorption at lower energies down to 1.25 eV due to sub-band-gap states (Figure S16a). We recorded time-resolved PL transients for the Cu_2_AgBiI_6_ thin films (Figure S16b) and fitted the decays to a stretched exponential function, yielding an average lifetime of 33 ns. This function phenomenologically accounts for a superposition of monoexponential decays,^53^ which may be a result of inhomogeneous trap distributions.^54^ Longer charge-carrier lifetimes are more favorable for photovoltaic applications, since they allow more time for the charge carriers to reach the contacts and be extracted but are sensitive to the trap density in the films and hence their processing conditions: for MAPbI_3_, monomolecular charge-carrier lifetimes ranging from 4 ns to over 1 μs have been reported.^54^ PL lifetime measurements of Cs_2_AgBiBr_6_ have also been made and are reproduced from Longo et al. in Figure S16b,^40^ and a stretched exponential was found to describe the long-term decays in this material as well, highlighting the heterogeneity of recombination processes in both Cs_2_AgBiBr_6_ and Cu_2_AgBiI_6_. Both materials have an initial fast PL decay, but Cu_2_AgBiI_6_ shows a higher proportion of signals from long-lived (>200 ns) PL in comparison to Cs_2_AgBiBr_6_, which is reflected in the lower average PL lifetime (10 ns) of the latter decay.

To gain an insight into charge-carrier mobilities in Cu_2_AgBiI_6_, we performed transient THz photoconductivity measurements using optical-pump, terahertz-probe spectroscopy, which gave a value for the electron–hole sum mobility of 1.7(5) cm^2^ V^–1^ s^–1^. This value is higher than that measured for the double perovskite Cs_2_AgBiBr_6_ (0.8 cm^2^ V^–1^ s^–1^),^55^ though not as high as that found in MAPbBr_3_ or in current best-in-class hybrid perovskites (8–70 cm^2^ V^–1^ s^–1^).^54,56^ Charge-carrier mobilities can be limited by intrinsic factors such as the effective mass of charge carriers and couplings of charge carriers to phonons but can also be influenced significantly by extrinsic factors such as crystallinity, energetic disorder, and carrier–carrier scattering.^54^ For instance, the first reports of room-temperature THz mobilities in CH_3_NH_3_SnI_3_ were only 1.6 cm^2^ V^–1^ s^–1^.^57^ However, through compositional tuning and more optimized processing, which resulted in reducing the crystalline disorder and background charge carrier density, this has been raised to over 80 cm^2^ V^–1^ s^–1^ for tin iodide perovskites.^58^ Mixed-cation, mixed-halide lead halide perovskites are similarly sensitive to extrinsic factors.^56^ Given the already-promising value for Cu_2_AgBiI_6_ measured here, an improved understanding of both the limits due to intrinsic factors and the influences of extrinsic factors could lead to further enhancements in charge-carrier mobilities in Cu_2_AgBiI_6_. Good charge-carrier diffusion lengths are critical to efficient solar cell operation, and a simplified calculation of , using the values measured here and neglecting higher-order recombination, yields an estimated value of 530 nm, which is greater than the first estimates for MAPbI_3_,^59,60^ showing a charge-carrier diffusion length suitable for charge extraction, despite the high cation disorder in the structure.

### 2.4 Cu_2_AgBiI_6_ Single-Junction Photovoltaic Devices

**Figure 4:**
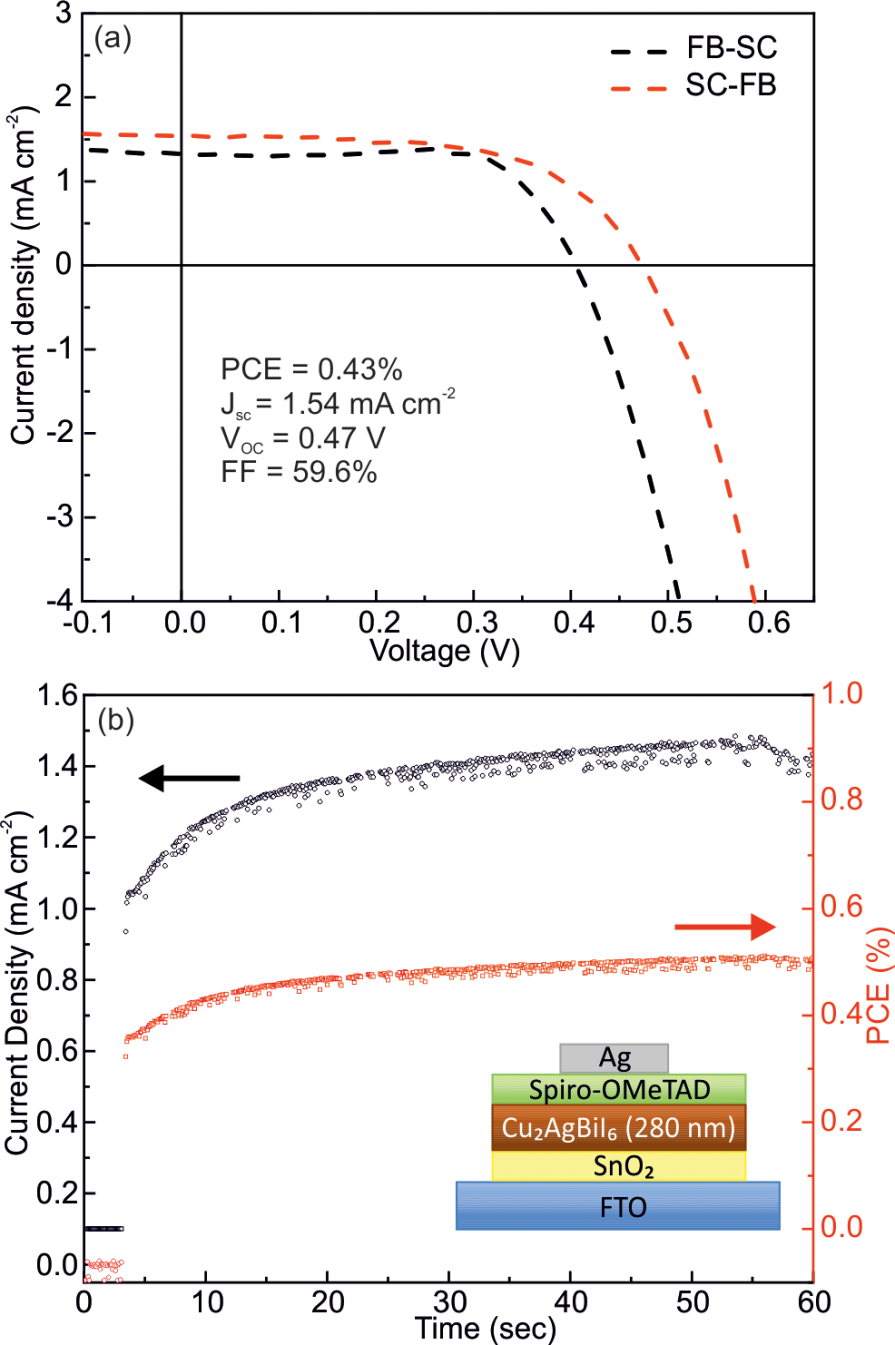
(a) *J*–*V* curves of the forward (FB) to short circuit (SC) and SC-FB scans for the device using Cu_2_AgBiI_6_ as the solar absorber. (b) Steady state performance, measured at the maximum power point, presenting good stability with both the current density and the PCE increasing over time. The inset shows the device architecture used.

In order to assess if this material does function as an absorber layer in a photovoltaic cell, we fabricated “n-i-p” planar heterojunction devices incorporating a compact SnO_2_ n-type charge extraction layer and a spiro-OMeTAD hole-extraction layer. We fully describe the cell preparation and measurements in the Supporting Information, with the device architecture being shown in the inset in Figure 4b. The cell did function and delivered a PCE of 0.43%, a *J*_sc_ of 1.54 mA/cm^2^, a *V*_oc_ of 0.47 V, and a fill factor of 59.6% (Figure 4a). The device shows hysteresis between the forward bias (FB)-to-short circuit (SC) and the SC-to-FB scan, with the first showing higher performance. However, it is interesting to note that the steady-state performances, measured at the maximum power point, present good short-term stability, with both the current density and the PCE increasing over time (Figure 4b). The results show that this device architecture can deliver photocurrent and photovoltage, but it is clear that significant effort is required to further optimize the devices. Here we have chosen the archetypical charge extraction materials and device configuration for lead halide perovskite cells, and it is likely that a new selection of charge extraction layers and/or different device architectures will be required in order to reach the full potential for this material. In addition, we expect that an improved understanding of the optoelectronic properties, and passivating defects, will also be important for device development.

We have demonstrated a certain degree of PV operation from this new material, yet the fundamental optical and electronic properties appear to promise significantly higher performance. Prior to expending significant effort upon materials and device optimization, however, it is important to estimate the ultimate potential efficiency achievable for this material. With knowledge of the above- and below-band-gap optical properties of a solar absorber material, it is possible to model its performance in a PV cell, following a detailed balanced thermodynamic approach,^52^ as we describe in the Supporting Information. In the thermodynamic assessment of a solar cell, Shockley and Queisser introduced an idealized step-function absorption profile, where the band gap is clearly defined.^61^ For a real material, the absorption onset is never infinitely steep, and the “PV band gap” is defined as the steepest point of the absorptance curve, which is easily deduced by taking the maximum of the differential of the external quantum efficiency spectrum. This PV band gap is therefore not an intrinsic property of the material but a property of the PV cell, which is influenced by the absorber layer thickness, its optical absorption properties, and the overall optical structure of the solar cell. In Figure S17b, we show the PV band gap of the Cu_2_AgBiI_6_ junction, as a function of the thickness of this layer. Very encouragingly, although the Elliott model band gap is 2.06(1) eV, the PV band gap drops from 2.0 eV all the way down to 1.7 eV for film thicknesses from 100 to 1200 nm. A band gap of 1.7 eV is close to optimal for combining with Si in a tandem cell, which will allow current matching of the two junctions.^11^ We therefore constructed an optical and electronic model for Cu_2_AgBiI_6_-on-Si tandem cells of the following structure: LiF/ITO/SnO_2_/ C60/Cu_2_AgBiI_6_/PolyTPD/ITO/nc-SiO_*x*_:H/(i)a-Si:H/c-Si/(i)a-Si:H/(p)a-Si:H/AZO/Ag (Figure S17a). We simulated the performance of a Cu_2_AgBiI_6_-on-Si tandem solar cell, using a transfer matrix optical model, coupled with a detailed balanced approach for simulating the current voltage curves. We determined the diode parameters for simulating the current–voltage curves via fitting lead halide perovskite and Si *J*–*V* curves reported in the literature (see the Supporting Information for more details and assumptions made during the modeling). Our main assumption is that, electronically, we can optimize the lead-free Cu_2_AgBiI_6_ to work as well as a lead halide perovskite cell, where radiative recombination accounts for 1% of the total recombination events (1% external radiative efficiency (ERE)), which is well below the world record lead halide perovskite cell that approaches 10% ERE but has not yet been reached in the related Ag_1–3*x*_Bi_1+*x*_I_4_ materials. Our model does account for the optical properties, including sub-band-gap absorption onset, of our experimentally measured Cu_2_AgBiI_6_ thin films. Our results suggest that, with a thickness of 1710 nm, the Cu_2_AgBiI_6_ thin film is capable of being the top cell in a Si tandem, yielding a matched current density of 19.0 mA/cm^2^ (Figure S17c), a *V*_oc_ of 1.92 V, an FF of 83%, and a corresponding PCE of 30.2% (Figure S17d). We show the influence of the decreasing Cu_2_AgBiI_6_ absorber layer thickness upon the tandem cell photovoltaic performance in Figure S18 in the Supporting Information. For a Cu_2_AgBiI_6_ layer thickness of 530 nm, the estimated carrier diffusion length leads to a modeled tandem efficiency of 28.1%. Thus, provided that the defects responsible for nonradiative recombination can be reduced in density or passivated to such an extent that a PV cell with a 1% external radiative efficiency can be created, then this material would compete on efficiency with lead halide perovskites integrated into multijunction PV cells. For comparison, we show in Figure S19 that Cs_2_AgBiBr_6_ cannot be current-matched with Si due to very low absorption in the red end of the visible spectrum.

## 3 Conclusion

In summary, we have synthesized stable compound Cu_2_AgBiI_6_ as powders, crystals, and solution-processed thin films. Cu_2_AgBiI_6_ provides the initial report and understanding of a quaternary phase in the CuI–AgI–BiI_3_ phase space. The structure is based on a 2D edge-sharing octahedral network. Octahedral sites are occupied by Ag^+^ and Bi^3+^ in a disordered fashion, and Cu^+^ occupies all possible tetrahedral sites located in the cubic close-packed iodide sublattice. Fitting the absorption profile using the Elliott model shows a band gap of the continuum of states of 2.06(1) eV and an exciton binding energy of only 25(2) meV. The steep rise in absorption from the band edge to a high absorption coefficient of 1.0 × 10^5^ cm^–1^ just above the onset, several times higher than for MAPbI_3_ (0.3 × 10^5^ cm^–1^), indicates great promise for the use of Cu_2_AgBiI_6_ as a thin-film absorber material in PV devices. In contrast, the highly studied lead-free double perovskite Cs_2_AgBiBr_6_ has a much less suitable absorption profile and exciton binding energy in comparison to Cu_2_AgBiI_6_ and a band gap too wide to be combined with c-Si in a tandem cell. The properties of this cation-decorated cubic close-packed iodide array containing Bi^3+^ emphasizes the scope for this chemistry to control optoelectronic properties without lead and beyond the perovskite structural family. Cu_2_AgBiI_6_ may also prove fruitful for other applications such as light emission and radiation detection.

## Supporting Information Available

Experimental and calculation details and characterization data (PDF)

## Accession Codes

CCDC 2013668 contains the supplementary crystallographic data for this paper. These data can be obtained free of charge via www.ccdc.cam.ac.uk/data_request/cif, or by emailing data_request@ccdc.cam.ac.uk, or by contacting The Cambridge Crystallographic Data Centre, 12 Union Road, Cambridge CB2 1EZ, UK; fax: +44 1223 336033.
