# Supplementary material for: Highly Absorbing Lead-Free Semiconductor Cu2AgBiI6 for Photovoltaic Applications from the Quaternary CuI–AgI–BiI3 Phase Space
Source: J Am Chem Soc. 2021 Mar 8;143(10):3983–92. doi: 10.1021/jacs.1c00495 (PMC8041282; doi:10.1021/jacs.1c00495)
Supplement: Supplementary file 1 — ja1c00495_si_001.pdf [file ja1c00495_si_001.pdf]

Highly Absorbing Lead-Free Semiconductor Cu_2AgBiI_6 for Photovoltaic Applications from the Quaternary CuI-AgI-BiI_3 Phase Space

Harry C. Sansom,[1,2] Giulia Longo,[2,†] Adam D. Wright,[2] Leonardo R.V. Buizza,[2] Suhas Mahesh,[2] Bernard Wenger,[2] Marco Zanella, [1] Mojtaba Abdi-Jalebi,[3,4] Michael J. Pitcher,[1,‡] Matthew S. Dyer,[1] Troy D. Manning,[1] Richard H. Friend,[4] Laura M. Herz,[2] Henry J. Snaith,[2] John B. Claridge,[1] Matthew J. Rosseinsky[1,*]

1. University of Liverpool, Department of Chemistry, Crown St., Liverpool L69 7ZD, U.K.
2. University of Oxford, Clarendon Laboratory, Department of Physics, Parks Road, Oxford OX1 3PU, U.K.
3. University College London, Institute for Materials Discovery, Malet Place, London WC1E 7JE, U.K.
4. University of Cambridge, Cavendish Laboratory, JJ Thomson Avenue, Cambridge CB3 0HE, U.K.

Present Addresses

† Northumbria University, Department of Mathematics, Physics and Electrical Engineering, Ellison Place, Newcastle upon Tyne NE18ST, U.K.
‡ CEMHTI CNRS UPR3079, 45071 Orléans, France

Methods

Cu_2AgBiI_6 powder and crystal synthesis

The starting materials used were CuI (99.999% Sigma–Aldrich) and AgI (99.999% Alfa Aesar). BiI_3 and $AgBiI_4$ were synthesised as previously reported.[1] Powder synthesis was carried out in evacuated sealed fused silica tubes. The fused silica tubes had a 6 mm internal diameter, 1 mm thick walls and were sealed at 10^{-4} mbar to 15 cm in length. The sample in the tube was cooled in liquid N_2 during the evacuation and sealing process to avoid sublimation of the iodides. CuI (0.0728g), AgI (0.0505g) and BiI_3 (0.1268g) powders (nominal composition $Cu_{1.85}Ag_{1.04}Bi_{1.04}I_{6.00}$, just off the $Cu_{4x}(AgBi)_{1-x}I_4$ solid solution line, were sealed in the evacuated fused silica tube. The tube was placed upright in a furnace and heated at 5°C/min to 610°C and kept there overnight before cooling at 5°C/min to 350°C where it was kept for 5 days. It was then cooled to room temperature at 5°C/min. The tube was scored and carefully broken open to retrieve the black powder, which was pelletised in to pellets 5 mm in diameter using a pellet press. The pellets were sealed in an evacuated fused silica tube. The second heating consisted of heating the powder up at 5°C/min to 350°C, keeping it there for 5 days before taking the tube out of the furnace and quenching the bottom half of it (containing the powder) to room temperature in a water bath. It was found to be crucial to quench the material from 350°C and keep the annealing temperatures below the melting point of the mixture to avoid a compositional inhomogeneity which spanned the $Cu_{4x}(AgBi)_{1-x}I_4$ solid solution line (Figures S1a and b). The tube was scored and carefully broken open to retrieve the black powder. A small crystal of approximate dimensions 20 µm × 30 µm × 10 µm, with an average SEM EDX composition of $Cu_{1.75(6)}Ag_{1.26(13)}Bi_{1.08(7)}I_{6.00(5)}$, was picked out of this sample for structural studies via SCXRD (Figure S2).

$CuBiI_4$ powder synthesis

A compositional screening of the $Cu_{3x}Bi_{1-x}I_3$ composition range was carried out between $0.18 \leq x \leq 0.32$, at 0.02 intervals (Figure S20). For this, CuI and BiI_3 powders were sealed in fused silica tubes. The sealed tubes were placed upright inside a furnace with enclosed heating elements and heated to 610°C to melt and mix the powders overnight. The furnace was cooled to 350°C at 5°C/min and kept there for 5 days. Afterwards, the tubes were quickly taken out of the furnace and the bottom half of each tube (containing the powder) was quenched to room temperature in a water bath. When the tubes were taken out of the furnace at 350°C and quenched, the red/purple vapour filling the tubes condensed above the water line away from the powder. The reactions formed a black powder at the bottom of the tube and tiny black crystals around the inside of the tubes. The small black crystals were too fragile to retrieve and would smear upon contact. The black powders were mechanically separated by carefully scoring and snapping open the tubes.

Cu_2AgBiI_6 thin film deposition

0.2418 g of AgI (1.03 mmol), 0.2381 g of CuI (1.25 mmol) and 0.5172 g of BiI_3 (0.877 mmol), corresponding to composition $Cu_{1.53}Ag_{1.26}Bi_{1.07}I_{6.00}$ were dissolved in 2 ml of a mixture of DMSO and DMF with a 3:1 volume ratio. The films were found to be consistently Cu-rich compared to the nominal composition in solution, showing loss of Ag ang Bi during the film processing. Therefore, the nominal composition reported here was to compensate for this, bringing the compositions of films more inline with the composition of the powders (Figure 2). The solution was stirred at 150°C for 30 minutes. The hot solution was filtered with a PTFE syringe filter with 0.22 µm pore size. The filtered solution was then kept at 75°C during the entire spin-coating process. The solution was spin-coated on different substrates in a N_2-filled drybox, with a speed of 4000 rpm for 60 seconds with 4000 rpm/s acceleration. The spin-coated films were then annealed at 50°C in air for 45 minutes, and, after this, at 150°C for 3 minutes. The films had a thickness of 280 nm, measured using a contact profilometer.

Cu_2AgBiI_6 device fabrication

Fluorine doped tin oxide (FTO) coated glass (15 Ω/sq) were etched using a 2M HCl solution and Zn powder. The patterned FTO substrates were cleaned through sequential sonication in soap, deionized water, acetone and isopropanol. The substrates were dried with a N_2 gun and exposed to O_2 plasma for 10 minutes. The SnO_2 electron transport layer was prepared by spin-coating a solution of $SnCl_4{\cdot}5H_2O$ in isopropanol (17.5 mg/ml) on top of the FTO coated glasses using 3000 rpm speed for 30 seconds. The so-prepared films were annealed at 100°C for 10 min followed by an annealing at 180°C for 30 min in air. After this the Cu_2AgBiI_6 film was deposited as explained in the thin film deposition. The hole transport material (SpiroOMeTAD, Lumtec) was dissolved in chlorobenzene (85 mg/ml) and doped with 20 µl of LiTFSI (500 mg/ml in butanol) and with 30 µl of tert-butylpyridine. This solution was then deposited on the Cu_2AgBiI_6 layer by dynamic spin-coating in air at 2000 rpm for 45 seconds. The devices were then left overnight in a dry air atmosphere, and then completed by the evaporation of 100 nm silver contacts. The device architecture is shown inset in Figure 4b. Device characterisations were performed in air unless specified otherwise.

Powder X-ray diffraction (PXRD)

PXRD data were measured on a Panalytical X'Pert Pro diffractometer using Co Kα_1 radiation (λ = 1.7890 Å) in Bragg–Brentano geometry and an X'Celerator detector. Phase identification was carried out using the X'Pert HighScore Plus (Version 2.2a)[2] with the PDF-2-ICDD database. PXRD data of capillaries used for photostability assessment were measured on a Bruker D8 Advance diffractometer using monochromated Mo Kα_1 radiation (λ = 0.7093 Å). TopasAcademic (Version 5) was used to perform Pawley fittings and Rietveld refinements of the data. VESTA[3] was used for graphical representation of the structures.

Single crystal X-ray diffraction (SCXRD)

Data were collected at 100K on a Rigaku MicroMax-007 HF diffractometer with a molybdenum rotating anode microfocus source and a Saturn 724+ detector using Rigaku Crystal Clear v2.0. Unit-cell indexation, data integration, and reduction were performed using Rigaku CrysAlisPro v171.38.43. The structure was solved and refined using SHELX-2013,[4] implemented through Olex2.[5]

Compositional Analysis

Scanning Electron Microscopy (SEM) and Energy Dispersive X-ray Spectroscopy (EDX) was used to measure the composition as a direct elemental analysis technique. Measurements were carried out using a Hitachi S-4800 SEM with an Oxford Instruments model 7200 EDS X-ray detector. Quantification was carried out using the microanalysis suite of the Inca Suite software (Version 4.15). All powders and crystals were sputtered with 15 nm Au to limit charging effects. Transmission Electron Microscopy (TEM) EDX was carried out using an JEOL JEM 2000FXII TEM microscope operating a W electron source operated at 200 kV, using an EDAX EDX detector, with quantification carried out using EDAX Genesis Spectrum (Version 5.217, 21-Jan-2008). All samples were prepared by spreading a finely ground powder onto carbon coated Au grids. Beam intensity had to be lowered, by increasing the spot size of the beam, as to not decompose the samples. All compositions calculated from EDX measurements were normalised to the nominal iodide content unless otherwise stated. The one standard deviation (1σ) spread in compositions measured for powder standards CuI and AgI, as well was the synthesised BiI_3, $CuBiI_4$ and Cu_2AgBiI_6 samples, are shown in Table S4.

Photostability

For the experiments, a Solar Light Model 16S-300-002 Solar Simulator was used which has a spectral output that complies with air mass 1.5 (AM1.5) per the ASTM standard definition. The combination of neutral density filters and lamp-to-sample distance allowed for the tuning of the intensity of the incident light to 1000 W m^{-2} as measured by a Solar Light Pyranometer PMA2144 and datalogging radiometer PMA2100. Sample temperatures were monitored using a T-type thermocouple and were found to stay below 35°C. For measurements in sealed atmospheres, powder was loaded into thin-walled (0.01 mm wall thickness) borosilicate capillaries under ambient air, dry synthetic air, and helium, and these were sealed with a gas-oxygen torch. The capillaries were then placed in the solar simulator and subjected to the full solar spectrum at an intensity of 1000 W m^{-2}.

Raman Spectroscopy

The measurements were carried out on a Renishaw inVia Reflex with a Leica microscope utilising a 633 nm wavelength red laser with a maximum power of 6.5 mW. The powder samples were measured in borosilicate capillaries and were exposed to 0.5% of the maximum laser power to avoid decomposition of the sample. The spot size was 5 µm**.**

Photoluminescence (PL) Spectroscopy

The Cu_2AgBiI_6 film was mounted in a gas-exchange helium cryostat (Oxford Instruments, OptistatCF2) and photoexcited by a 398 nm picosecond pulsed diode laser (Picoquant, LDH-D-C-405M). The resultant PL was collected and coupled into a grating spectrometer (Princeton Instruments, SP-2558), which directed the spectrally dispersed PL onto a photon-counting detector (PDM series from MPD), whose timing was controlled with a PicoHarp300 TCSPC event timer. A laser fluence of 200 nJ cm^{-2} was used for both the spectral and transient measurements of Cu_2AgBiI_6, which were both taken at a temperature of 295 K. The PL decay trace in Figure S16b was measured at a wavelength of 720 nm. The PL decay trace was fitted by a stretched exponential function $I = I_0 \exp(-(t/\tau)^{\beta})$, where β is the distribution coefficient and τ is the time taken for the PL intensity to drop to I_0/e. Such stretched exponential functions have been used to phenomenologically account for the presence of a local distribution of monoexponential decay rates, whose average lifetime is given by $\tau_{av} = (\tau/\beta)\ \Gamma(1/\beta)$, where Γ is the gamma function.[6-7]

The PL spectrum for $MAPbI_3$ in Figure S15 was measured using the same experimental setup, except it was mounted in a cold-finger cryostat (Oxford Instruments, MicrostatHe) and detected with an iCCD (PI-MAX4, Princeton Instruments), under a laser excitation fluence of 490 nJ cm^{-2}.The spectrum was previously published in Wright *et al*.[8]

UV-Visible absorption measurements using FTIR spectrometer

The UV-Visible absorption spectra in Figure 3 in the main text were measured using a Bruker Vertex 80v Fourier transform infrared (FTIR) spectrometer, configured with a tungsten halogen lamp illumination source, a CaF_2 beamsplitter and a silicon detector. The samples were mounted in a gas-exchange helium cryostat (Oxford Instruments, OptistatCF2). The $MAPbI_3$ absorption spectrum was previously published in Davies *et al*.[9]

THz photoconductivity

An amplified laser system (Spectra Physics, MaiTai–Empower–Spitfire) with a central wavelength of 800 nm, 35 fs pulse duration and 5 kHz repetition rate was used to generate THz radiation via the inverse spin Hall effect, using an emitter made of 2 nm of tungsten / 1.8 nm of $Co_{40}Fe_{40}B_{20}$/ 2 nm of platinum, supported by a quartz substrate. The transmitted THz radiation was detected using free-space electro-optic sampling with a 1 mm thick ZnTe (110) crystal, a Wollaston prism and a pair of balanced photodiodes. The THz pulse was measured in transmission geometry. The pump beam was frequency-doubled to 400 nm by a β-barium-borate (BBO) crystal. Charge-carrier mobilities

were calculated from the initial transmitted signal at time = 0 ps at fluences of 7.8, 15.3 $\mu J cm^{-2}$, as outlined in Wehrenfennig *et al.*,[10] and measurements were carried out under vacuum ($< 10^{-2}$ mbar).

J–V characterisation

J–V characterisation was measured using a Keithley 2400 sourcemeter and simulated air-mass 1.5 global tilt (AM1.5G) solar irradiation using a Wavelabs Sinus-220 light-emitting diode array, calibrated with a certified Si reference cell. The areas being measured were defined by using a black anodised aluminium mask placed directly in contact with glass side of the substrate and an enclosed sample holder, to shadow the rest of the device.

Photothermal deflection spectroscopy (PDS)

PDS is an ultrasensitive absorption measurement technique that detects heating of the sample due to the non-radiative relaxation of absorbed light and is insensitive to reflection and scattering. PDS enables the detection of absorbance signals with 5–6 orders of magnitude weaker than the band edge absorption. For the measurements, a monochromatic Pump light beam is shined on the sample (film on Quartz substrate), which on absorption produces a thermal gradient near the sample surface via non-radiative relaxation induced heating. This results in a refractive index gradient in the area surrounding the sample surface. This refractive index gradient is further enhanced by immersing the sample in an inert liquid FC-72 Fluorinert® (3M Company) which has a high refractive index change per unit change in temperature. A fixed wavelength CW laser probe beam is passed through this refractive index gradient producing a deflection proportional to the absorbed light at that particular wavelength, which is detected by a photo-diode and lock-in amplifier combination. Scanning through different wavelengths gives us the complete absorption spectra. Because this technique makes use of the non-radiative relaxation processes in the sample, it is immune to optical effects like interference and scattering. Furthermore, PDS technique is a powerful technique to measure the sub-bandgap tail states in a semiconductor up to an absorption coefficient of 1 cm^{-1}.

Elliot Model Fitting

We use Elliott's formula[11] which expresses the absorption coefficient as a linear combination of the absorption of a bound exciton with the absorption by the joint continuum of states[9, 12]:

$$\alpha(E) = \alpha_x(E) + \alpha_{cont}(E)$$

with

$$\alpha(E) = \frac{2(2\mu)^{\frac{3}{2}}e^2}{ncm_0^2\hbar^3}\frac{|P_{cv}|^2}{E}\left[\sum_{n=1}^{\infty}\frac{4\pi E_x^{3/2}}{n^3}\delta\left(E-\left(E_g-\frac{E_x}{n^2}\right)\right)+\frac{2\pi\sqrt{\frac{E_x}{E-E_g}}}{1-\exp\left(-2\pi\sqrt{\frac{E_x}{E-E_g}}\right)}\Theta\left(\sqrt{E-E_g}\right)\right]$$

Where *E* is the photon energy, E_x is the exciton binding energy, E_g is the band gap, *n* is the refractive index (at energy E), $|P_{cv}|^2 = |\langle\Psi_c|P|\Psi_v\rangle|^2$ is the momentum matrix element, δ is the Dirac delta function, $\Theta(x)$ is the step function, μ is the reduced mass, m_0 is the electron rest mass, *e* is the elementary charge, *c* is the speed of light in vacuum and $\hbar$ is the reduced Planck's constant.

The experimental absorption spectrum is fitted with the following model:

$$\alpha(E) = \frac{A}{E}\ [(\alpha'_x * g_x)(E) + (\alpha'_{cont} * g_{cont})(E)\ ]$$

Where A is a fitting constant and the excitonic part of the absorption (α'_x) and the continuum part (α'_{cont}) are convoluted with gaussian broadening functions g_x and g_{cont}, respectively.

Optical Modelling

The generalised transfer matrix method was used to model the optical response of the stack.[13] The python libraries Numpy and Scipy were used to perform the calculations. Transfer matrix calculations take the complex refractive index spectrum and thickness for each layer as input. The calculation provides us with absorptance of each layer, and the transmittance and reflectance of the stack. We assumed perfect internal quantum efficiency and calculated the short circuit current J_{sc} as the overlap integral of the AM1.5 solar spectrum with the absorptance. The JV curve of each sub-cell was modelled as a single diode: $J = J_{sc} - J_0 e^{\frac{qV}{nkT}}$. Here, n is the ideality factor. The recombination current J_0 is calculated through the principle of detailed balance: $J_0 = \int_0^\infty EQE_{PV}(E)\cdot\phi_{BB,300K}(E)\cdot dE$. Here $\phi_{BB,300K}(E)$ is the blackbody photon flux at 300K, and $EQE_{PV}(E)$ is the absorptance calculated from the transfer matrix calculation. The following diode parameters were assumed for the Cu_2AgBiI_6 sub-cell:[14] $R_{Shunt} = 5\ k\Omega\cdot cm^2$, $R_{Series} = 4.2\ \Omega\cdot cm^2$, n =1.0, $EQE_{EL} = 0.01$. The calculated JV parameters for the Cu_2AgBiI_6 sub-cell were: PCE = 18.1%, V_{OC} = 1.16 V, J_{SC} = 19.0 mA/cm^2, FF = 0.82. The following were

assumed for the Si sub-cell:[15] $R_{Shunt,Si} = 10\ k\Omega \cdot cm^2$, $R_{Series,Si} = 0.4\ \Omega \cdot cm^2, n_{Si}$ =1.04, $EQE_{EL,Si} = 0.0056$. The calculated JV parameters for the Silicon sub-cell were: PCE = 12.1%, V_{OC} = 0.75 V, J_{SC} = 19.0 mA/cm^2, FF = 0.84. The thickness of the LiF (20-200nm) the two ITO layers (20-200nm), and Cu_2AgBiI_6 (1200−1800nm) were varied with the indicated bounds using a differential evolution algorithm till a PCE maximum was obtained. The stack used as input for the Transfer Matrix Calculations is given in Table S5. The source of optical constants for each layer are also cited. For Cu_2AgBiI_6, a "synthetic" absorption co-efficient was created by splicing a 74 meV tail (gradient 1/0.074 eV) between 1.0 eV−1.88 eV, extracted from the gradient of the absorption onset from the combined PDS and FTIR datasets, to the absorption co-efficient obtained from FTIR measurements. The absorption co-efficient was set to zero below 1.0 eV. This was then converted into the extinction coefficient (Figure S21) which was transformed into the refractive index using the Kramer's Kronig relation:[16] $n(\lambda) = 1 + \frac{2}{\pi}\int_0^\infty \frac{E'k(E)}{E'^2 - E^2} dE'$. The described method assumes the following:

1. Transfer Matrix Model Limitation: Layer roughness is much smaller than the wavelength of light (~550nm).
2. The "synthetic" absorption coefficient created by appending a tail of energy 74 meV between 1.88-1.45 eV characterises the material well.
3. The same transport materials conventionally used for hybrid perovskites, C60/SnO_2 and PolyTPD are used. This is a relatively minor assumption as most transport layers are thin and poorly absorbing in comparison to the absorber layer, so the precise transport layer does not matter.
4. The Cu_2AgBiI_6 sub-cell can be optimised to bring it to the same radiative efficiency (EQE_{EL} = 1%) as a well-performing hybrid lead halide perovskite cell. The same shunt and series resistances ($R_{Shunt} = 5\ k\Omega \cdot cm^2$, $R_{Series} = 4.2\ \Omega$) can also be achieved.

Density Functional Theory Calculations

All periodic density functional theory calculations were performed using version 5.4 of the the VASP code[17] with the projector augmented wave method to describe core electrons.[18] The crystal structure prediction package ChemDASH[19] was used to generate low energy ordered configurations of atoms within (2*a*+*b*, *a*+2*b*, *c*) supercells of the disordered experimental structure. Four independent starting configurations were initially generated by randomly occupying the experimental mixed and partially occupied Bi, Ag and Cu sites within the cell. ChemDASH was then used to generate 249 further configurations from each starting configuration by swapping between Bi, Ag and vacancies on the octahedral sites, and Cu and vacancies on the tetrahedral sites. A basin hopping approach using a value for k_BT of 0.01 eV/atom for the Metropolis acceptance criterion was used to ensure that low energy configurations were generated. The geometry of each configuration was optimised using the optB86b-vdW functional[20] to model the important van der Waals interactions between the large iodine anions within the structure. ChemDASH uses a multi-step approach to geometry optimisation, where the final step used here had a plane-wave energy cutoff of 500 eV and forces were minimised to below 0.02 eV/Å. The lowest energy configuration of the 249 generated starting from each of the four starting configurations were then taken forwards to calculate the electronic structure, giving four low energy configurations with significantly different Bi, Ag and Cu orderings. A more accurate geometry was obtained for each of these configurations by performing a further geometry optimisation with the optB86b-vdW functional but with a higher plane-wave energy cutoff of 550 eV and a denser *k*-point mesh with a *k*-point spacing of 0.1 Å^{-1} and to a tighter force threshold of 0.001 eV/Å for convergence (Figure S22). A single-point calculation using the SCAN meta-GGA functional[21] and including the effects of spin-orbit coupling was then used to compute the electronic structure of each of these four configurations, resulting in the partial density of states plots shown in Figure 3c and Figure S13. Band structure diagrams in Figure S14 for the lowest energy computed structure of Cu_2AgBiI_6 utilise the SCAN functional and including spin-orbit coupling effects

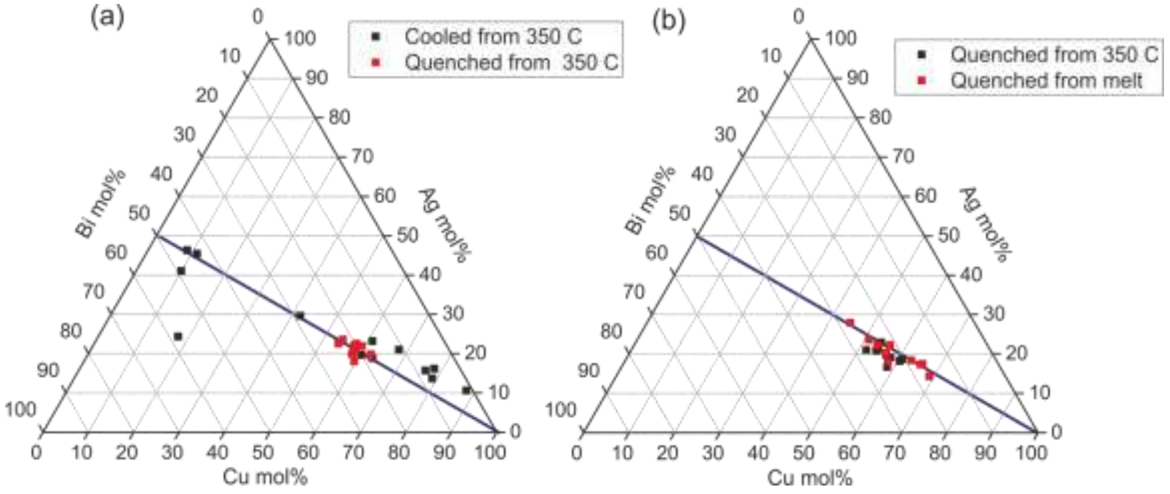

Figure S1. (a) The TEM EDX of particles from the Cu_2AgBiI_6 powder synthesis, showing how quenching from 350°C, rather than cooling slowly to room temperature, gives a compositionally homogeneous sample. (b) The samples are also more compositionally homogeneous if the melt is avoided.

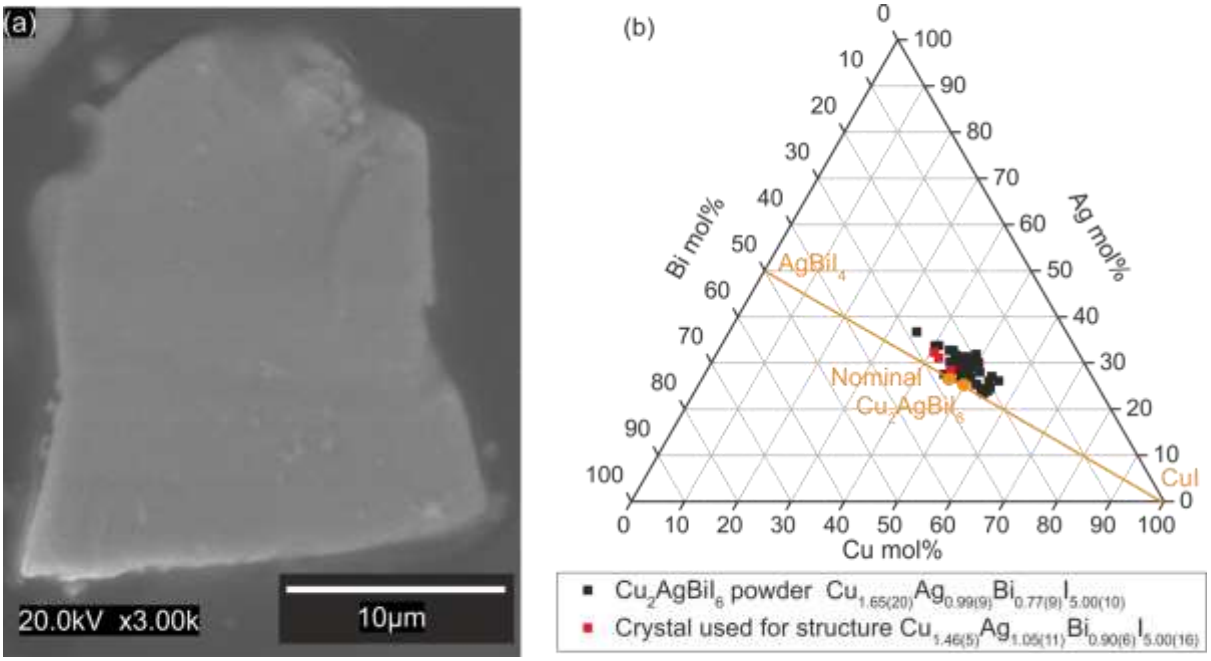

Figure S2. (a) The SEM image of the Cu_2AgBiI_6 crystal used for the SCXRD structural solution. The crystal was picked out of the Cu_2AgBiI_6 powder synthesis. (b) The SEM EDX showing the composition of the crystal to be within error of the powder.

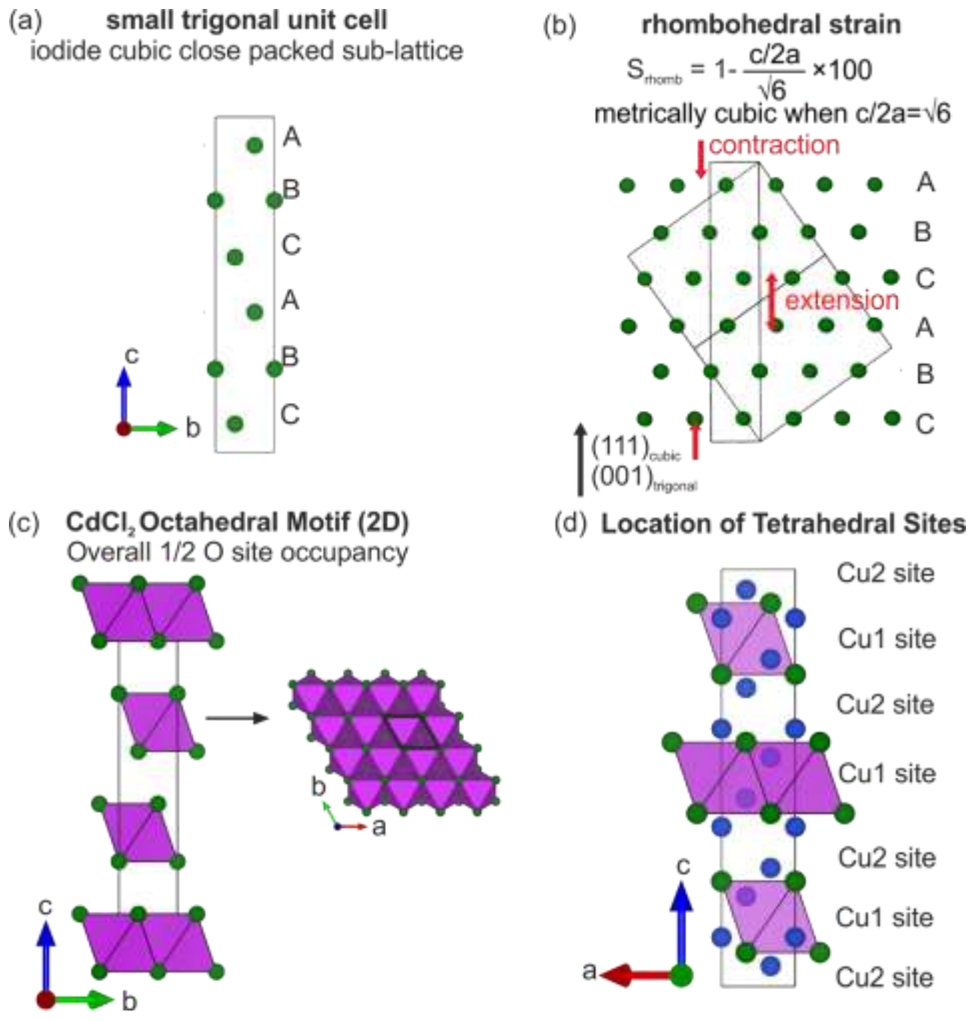

Figure S3. (a) The trigonal unit cell and cubic close packed (CCP) iodide sub-lattice of Cu_2AgBiI_6 (b) The quantitative definition of rhombohedral strain used here, as well as the visualisation of the relationship between a metrically cubic trigonal cell ($R\bar{3}m$) and a cubic unit cell ($Fd\bar{3}m$). (c) The octahedral motif of Cu_2AgBiI_6 (the $CdCl_2$ structure). The number of octahedral sites occupied is ½ of the total allowed by the structure (due to the vacant layers), however in Cu_2AgBiI_6, the atomic occupancies of these sites are less than one (disordered). (d) The two tetrahedral sites of Cu_2AgBiI_6. Site Cu1 is found in the layers containing octahedral occupancy, and site Cu2 is found in the layers without octahedral occupancy. In Cu_2AgBiI_6, these Cu sites are not fully occupied (disordered) but do show layered ordering and therefore can be described as possessing partial layered ordering.

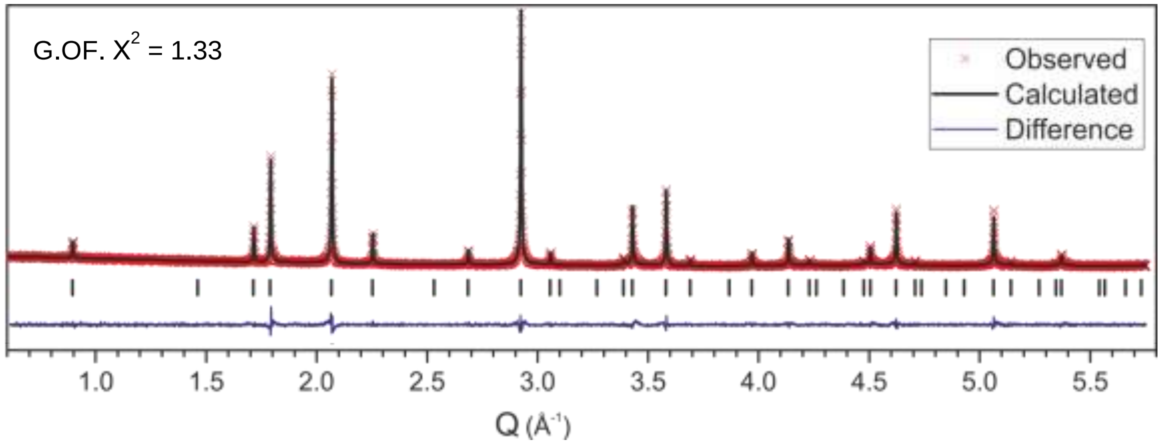

Figure S4. The Pawley fit to room temperature laboratory PXRD data of $CuBiI_4$. The PXRD pattern is fitted using a cubic unit cell with space group Fd$\overline{3}$*m*, previously reported by Fourcroy *et al.*[22] The extracted lattice parameter is *a* = 12.1580(2) Å.

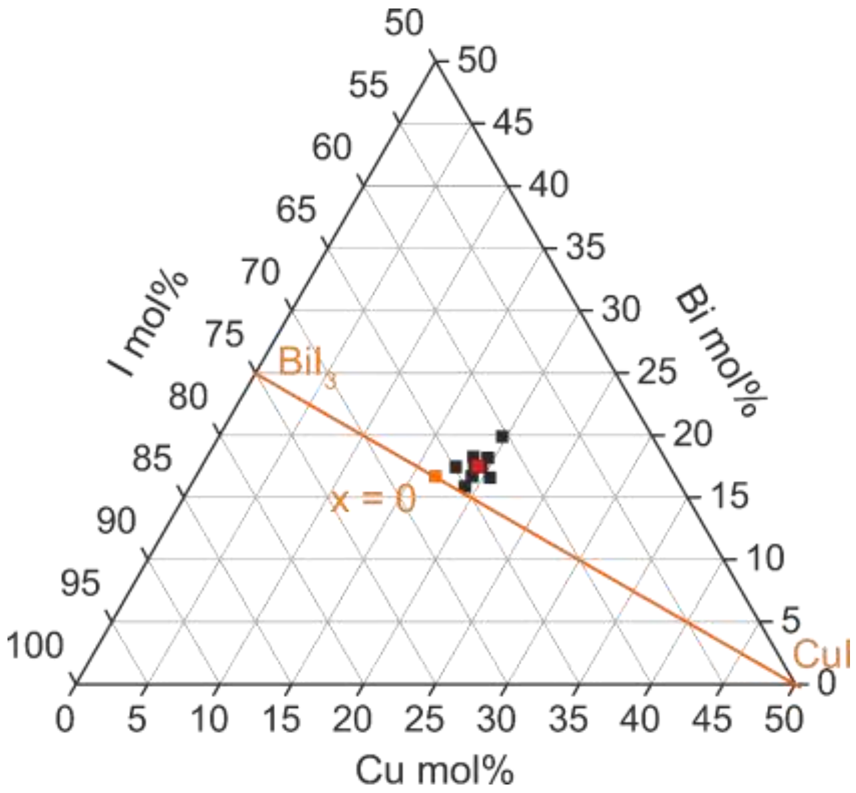

Figure S5. SEM EDX of the powder corresponding to nominal composition *x* = 0 $Cu_{1\text{-}3x}Bi_{1+x}I_4$, giving an average composition of $Cu_{1.21(5)}Bi_{1.11(7)}I_{4.00(9)}$, and referred to as $CuBiI_4$ throughout the text.

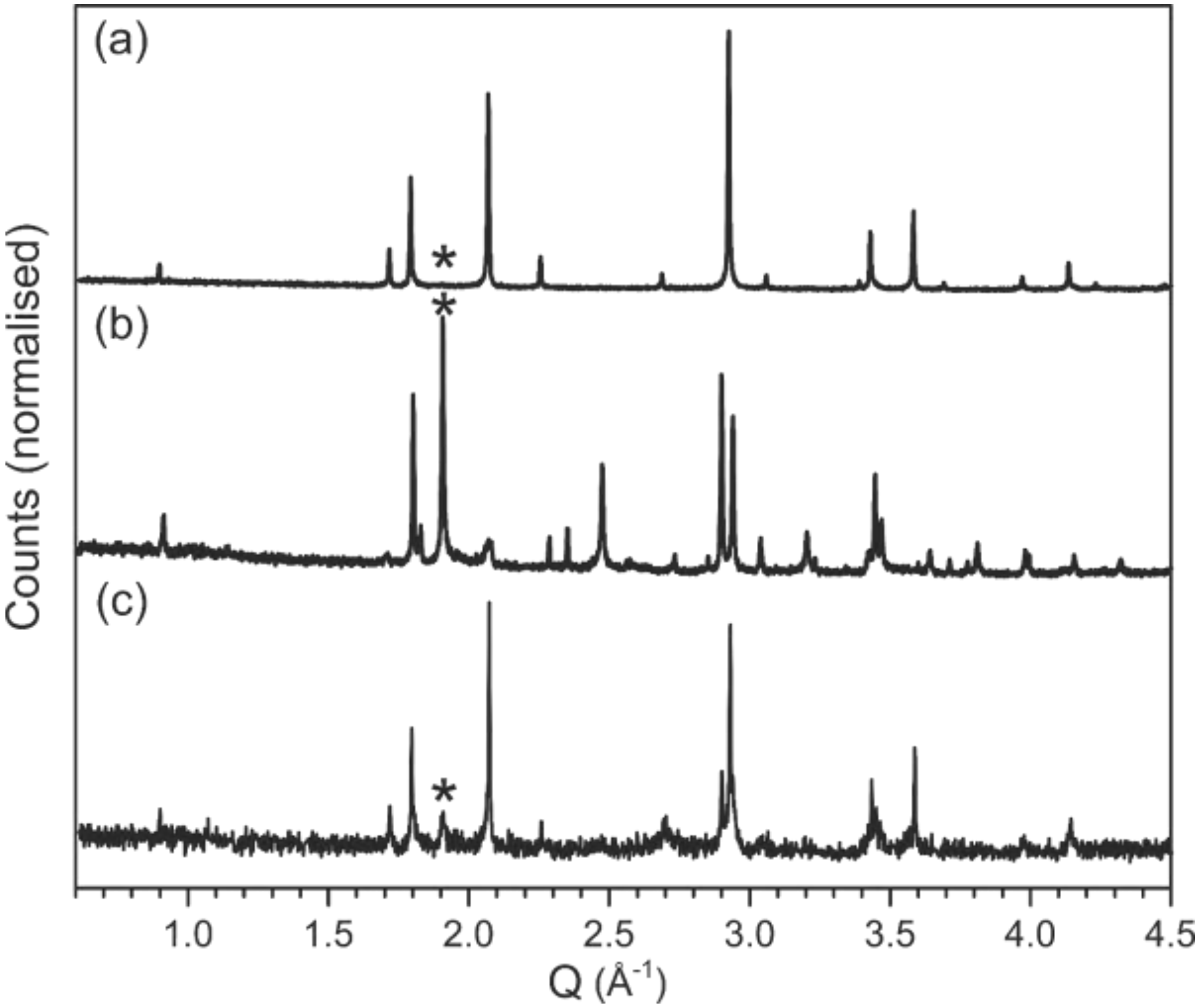

Figure S6. The PXRD patterns of (a) $CuBiI_4$ as synthesised (b) $CuBiI_4$ kept in the dark in air at room temperature for three weeks, and (c) $CuBiI_4$ kept in the dark at -20°C for three weeks. The main peak of the BiI_3 decomposition phase is marked by the asterisk.

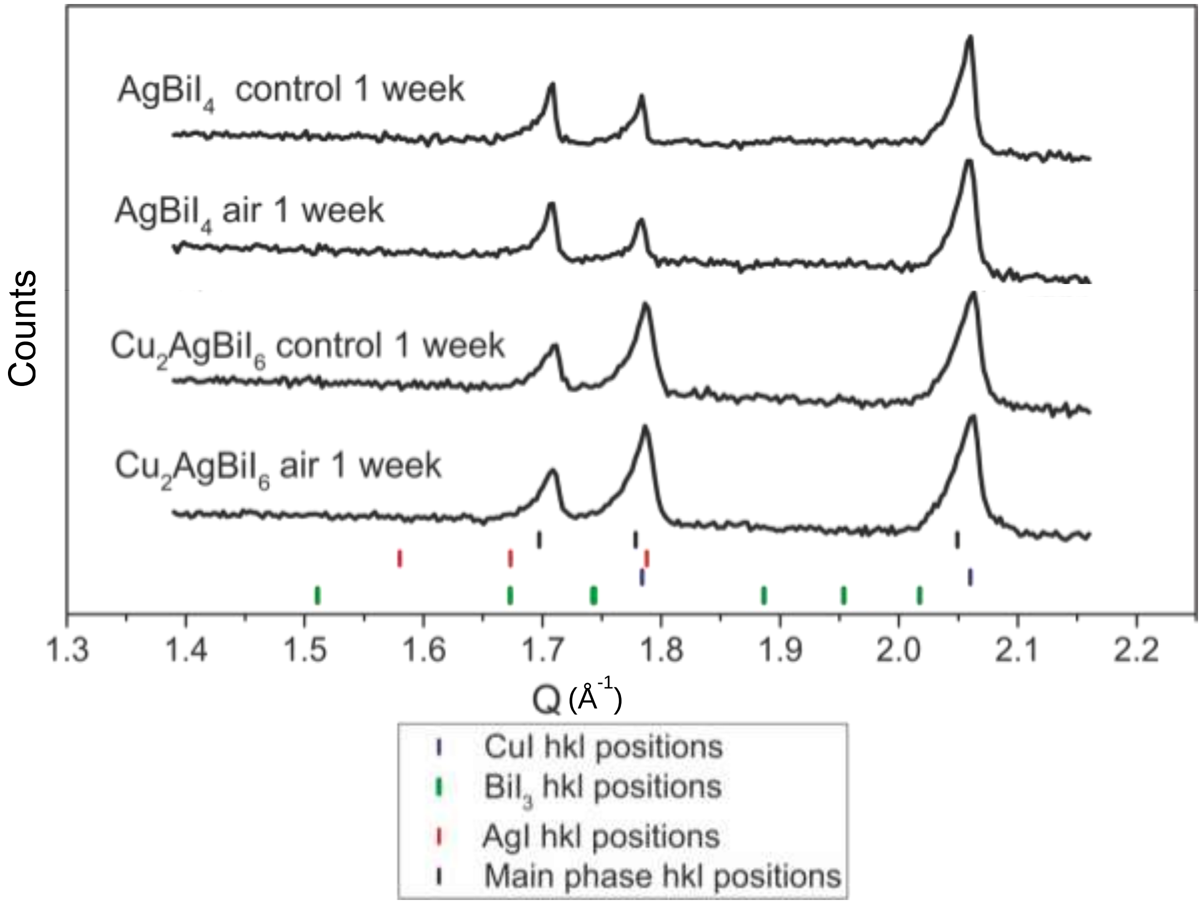

Figure S7. The PXRD patterns of $AgBiI_4$ and Cu_2AgBiI_6 powders after being exposed to one week in the solar spectrum, sealed in capillaries in air. Also shown are the PXRD of the controls, which were kept in the dark in air. The range shown includes where the largest peaks would appear for possible decomposition phases AgI (black tick marks), CuI (red tick marks) and BiI_3 (blue tick marks), should they have been present.

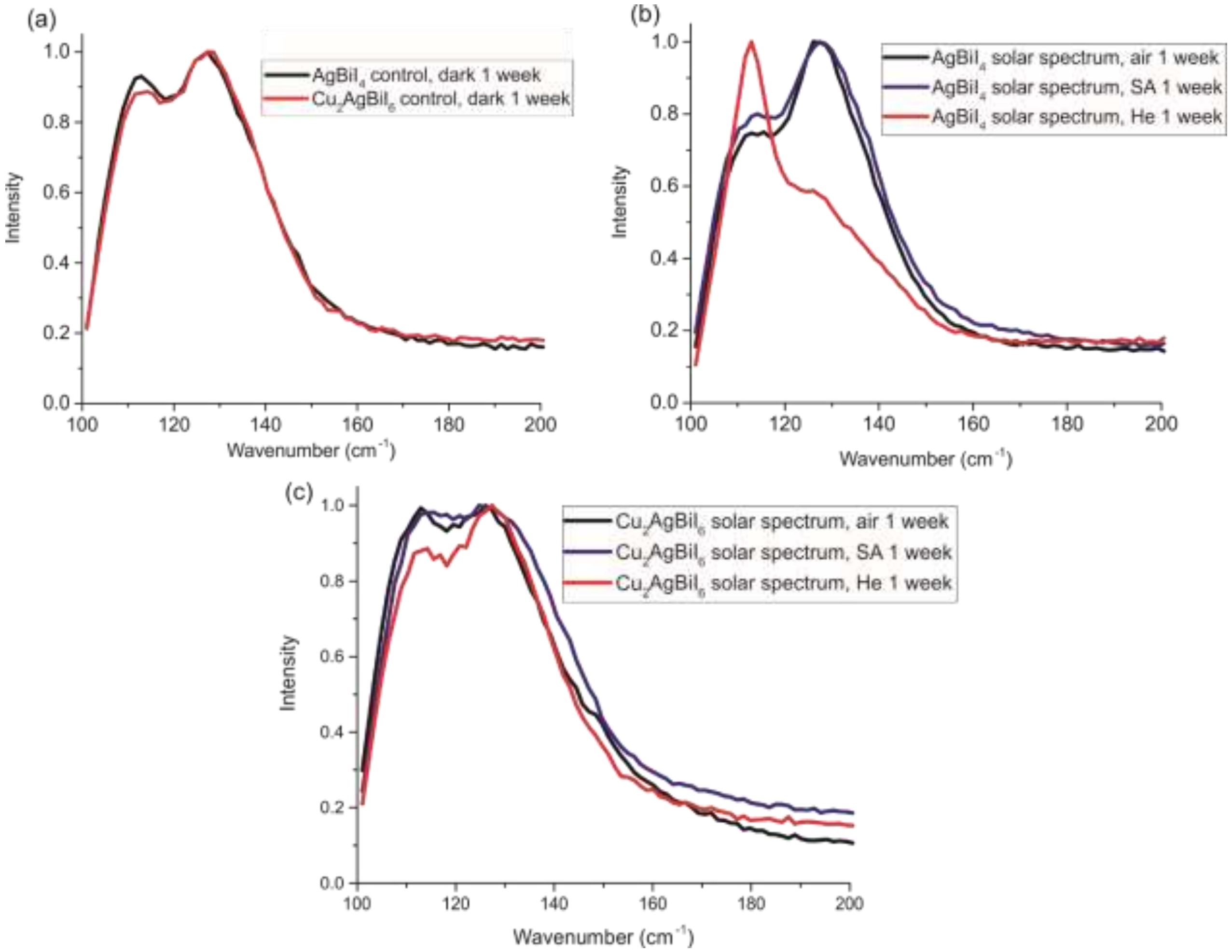

Figure S8. (a) The Raman spectra of $AgBiI_4$ and Cu_2AgBiI_6 control samples, showing the characteristic two peaks, which occur at low wavenumbers. The Raman spectra of $AgBiI_4$ and Cu_2AgBiI_6 powders after being exposed to the AM1.5 solar spectrum in different atmospheres are shown in b and c, respectively. There are no significant changes in the spectra of either materials. The change in ratio of the two peaks in the $AgBiI_4$ He 1 week spectra is likely due to the disorder of the Ag^+ and Bi^{3+} cations.

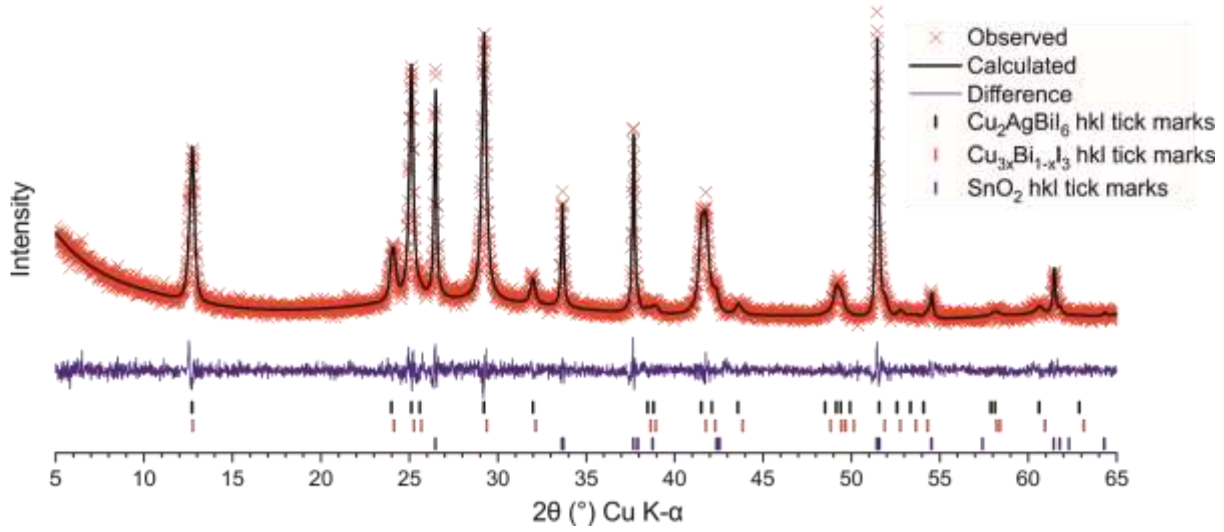

Figure S9. Pawley fit of PXRD pattern for Cu_2AgBiI_6 films spincoated on FTO glass, fitted to a rhombohedral phase associated with a quaternary phase (black tick marks), a rhombohedral phase consistent with a $Cu_{3x}Bi_{1-x}I_3$ phase (red tick marks), and SnO_2 (blue tick marks).

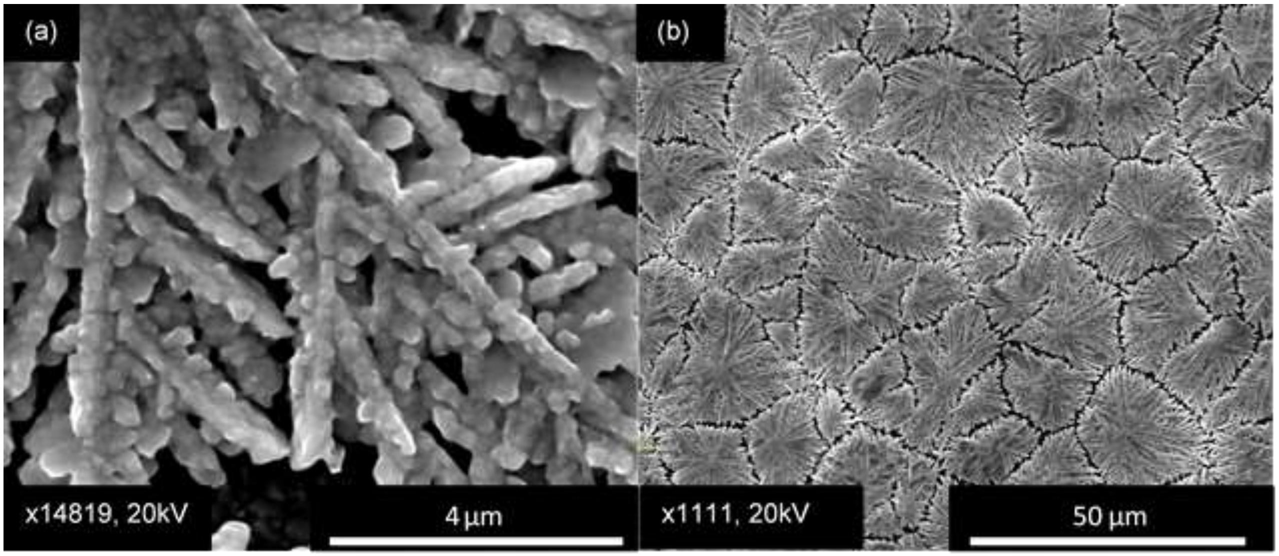

Figure S10. SEM images of (a, b) rough, large, dendritic grains obtained during the early stages of solution processing the Cu_2AgBiI_6 films.

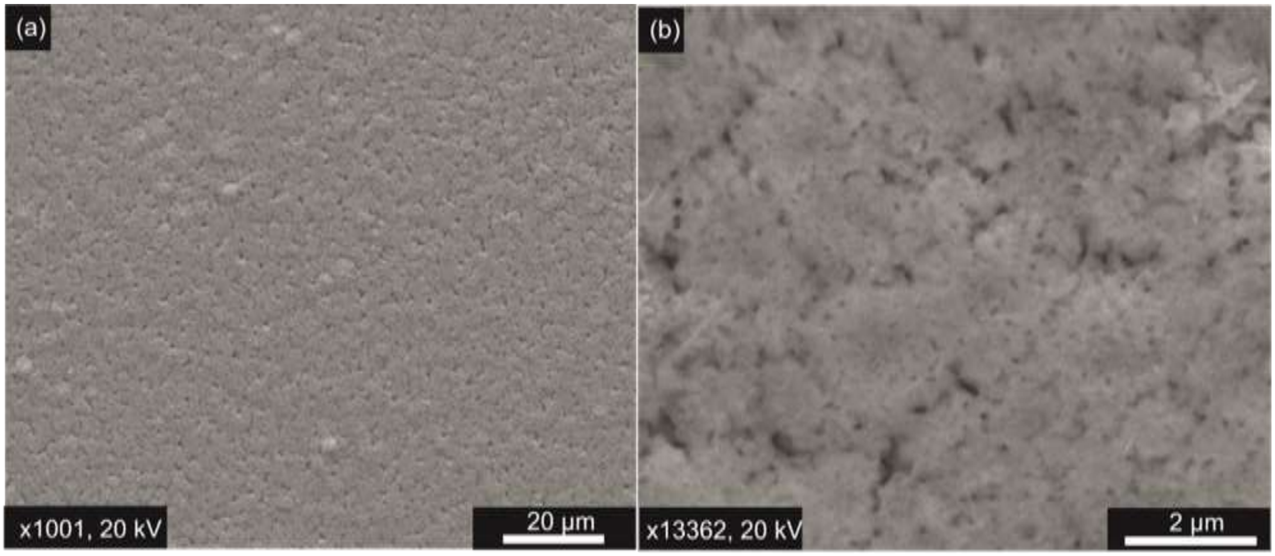

Figure S11. (a, b) SEM images of the uniform, smooth morphology of the optimised Cu_2AgBiI_6 film at x1001 and x13362 magnification, respectively

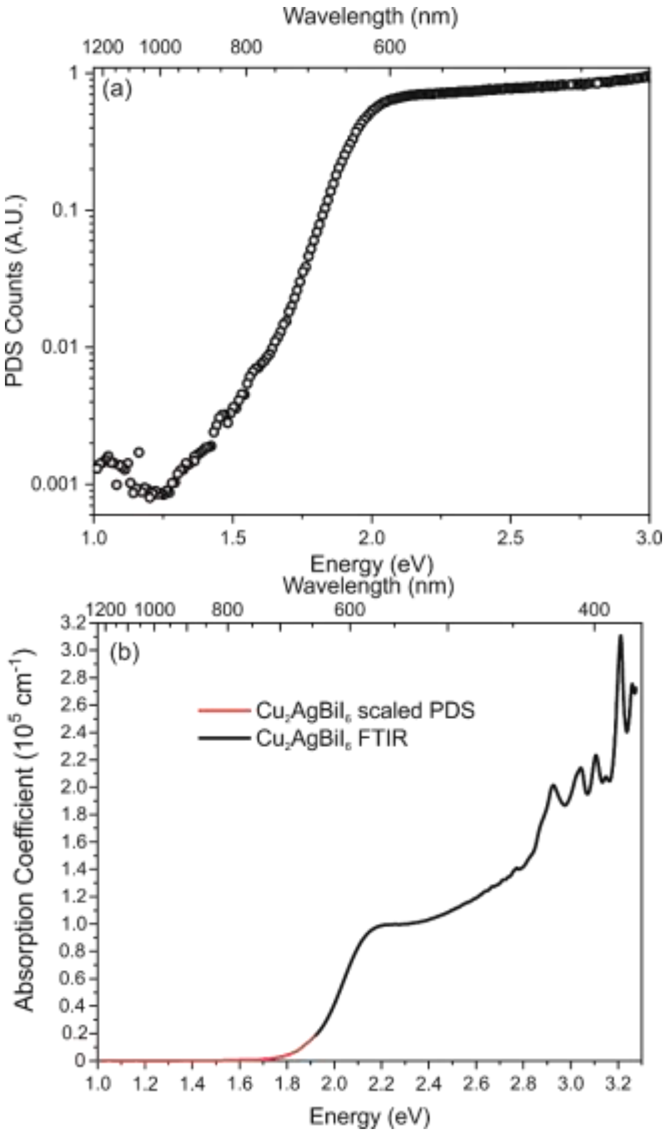

Figure S12. (a) The raw PDS data of Cu_2AgBiI_6 thin films. (b) The PDS data (red) is scaled to match the absorption coefficient measured by FTIR (black).

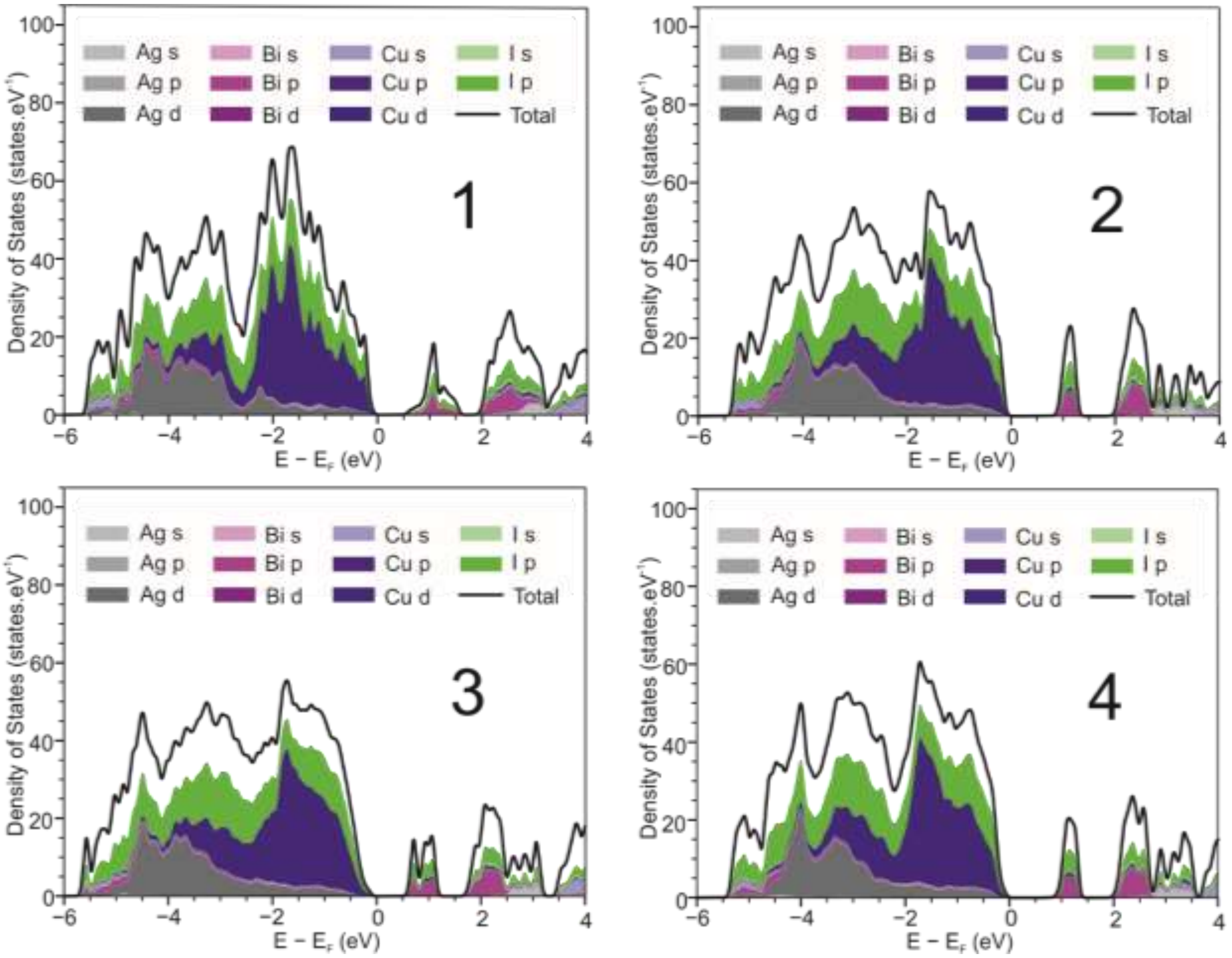

Figure S13. The partial density of states plots for the lowest energy configurations arising from each of the four independent ChemDASH calculations shown in Figure S22. The electronic structure is computed using the meta-GGA functional, SCAN,[21] and including spin-orbit coupling. Shown on each plot is the value of the smallest gap between occupied and unoccupied bands. The plot for configuration 1, the lowest energy configuration overall is also shown in Figure 3c of the main text, but is included again here for comparison. Although changing the configuration of Ag^+, Bi^{3+} and Cu^+ cations within the computed cell does affect the position and shape of peaks in the density of states, in all configurations the Cu 3*d*-states dominate at the valence band edge, and the conduction band consists of mixed Bi 6*p*-states and I 5*p*-states.

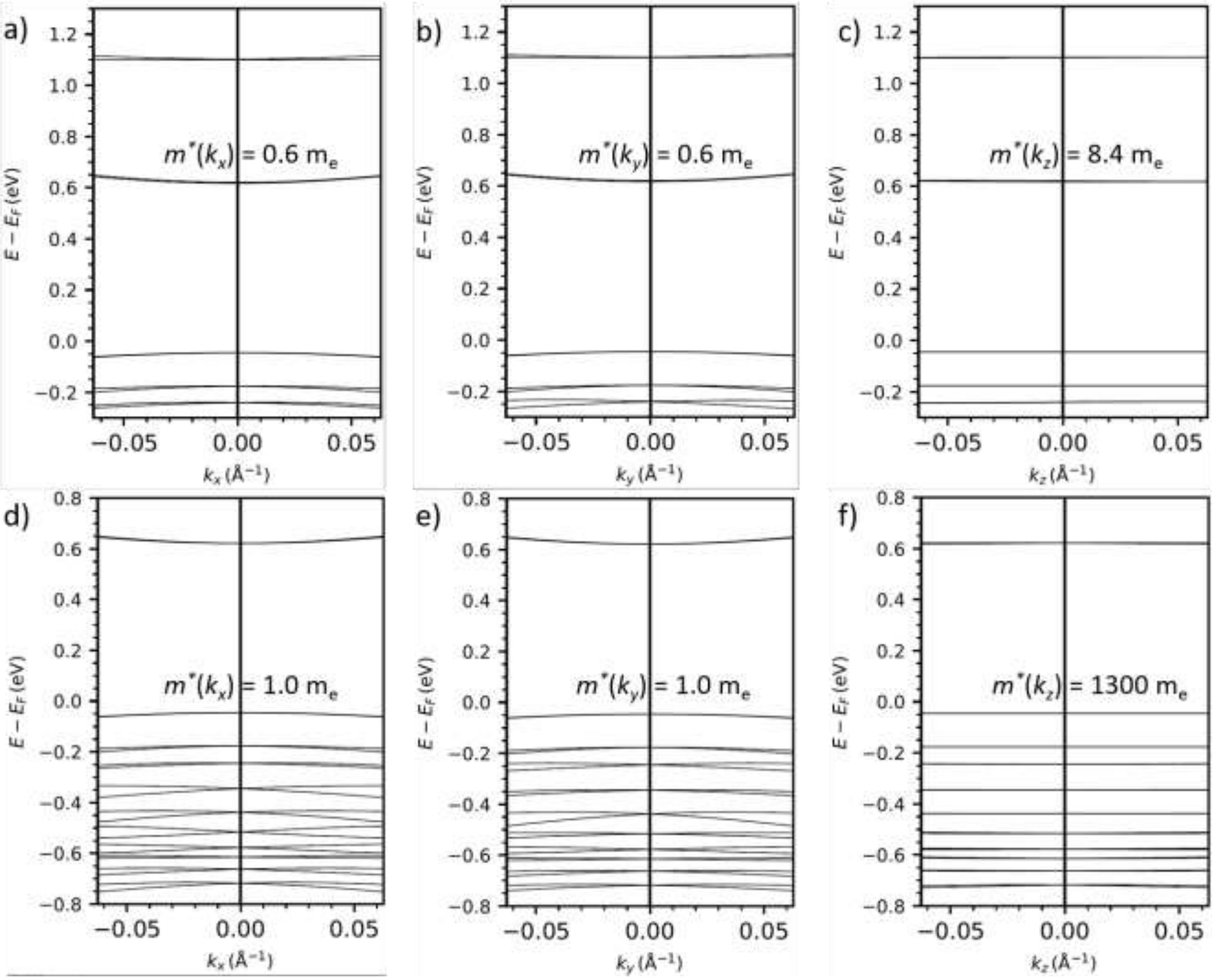

Figure S14. Band structure diagrams for the lowest energy computed structure of Cu_2AgBiI_6 (Figure S22) using the SCAN functional and including spin-orbit coupling effects. a)-c) Show the band dispersion around the conduction band maximum in the k_x, k_y and k_z directions and the effective mass of electrons derived from these. d)-f) Show the band dispersion around the valence band maximum in the k_x, k_y and k_z directions and the effective mass of holes derived from these.

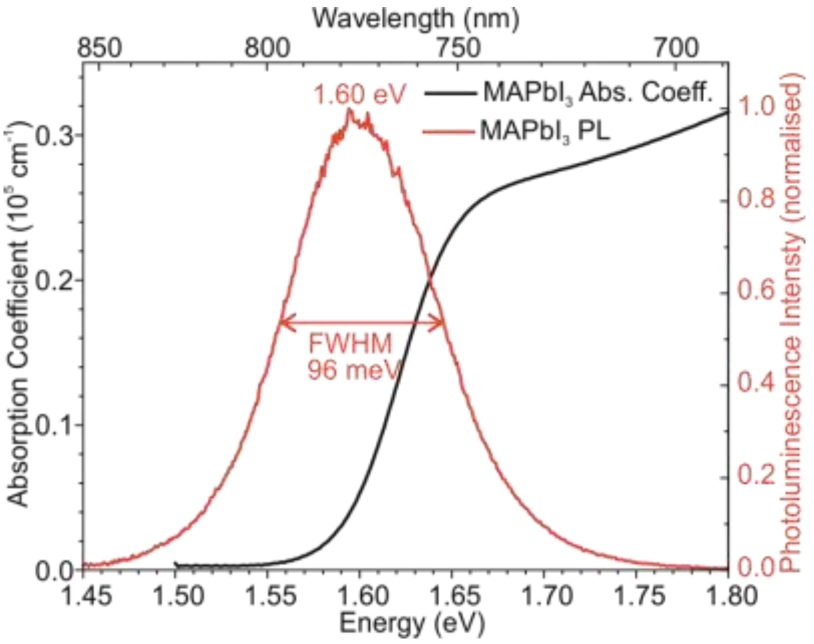

Figure S15. The reported photoluminescence (red) and absorption coefficient (black) of $MAPbI_3$ thin films, reproduced from Wright *et al*. and Davies *et al.* respectively. [8-9]

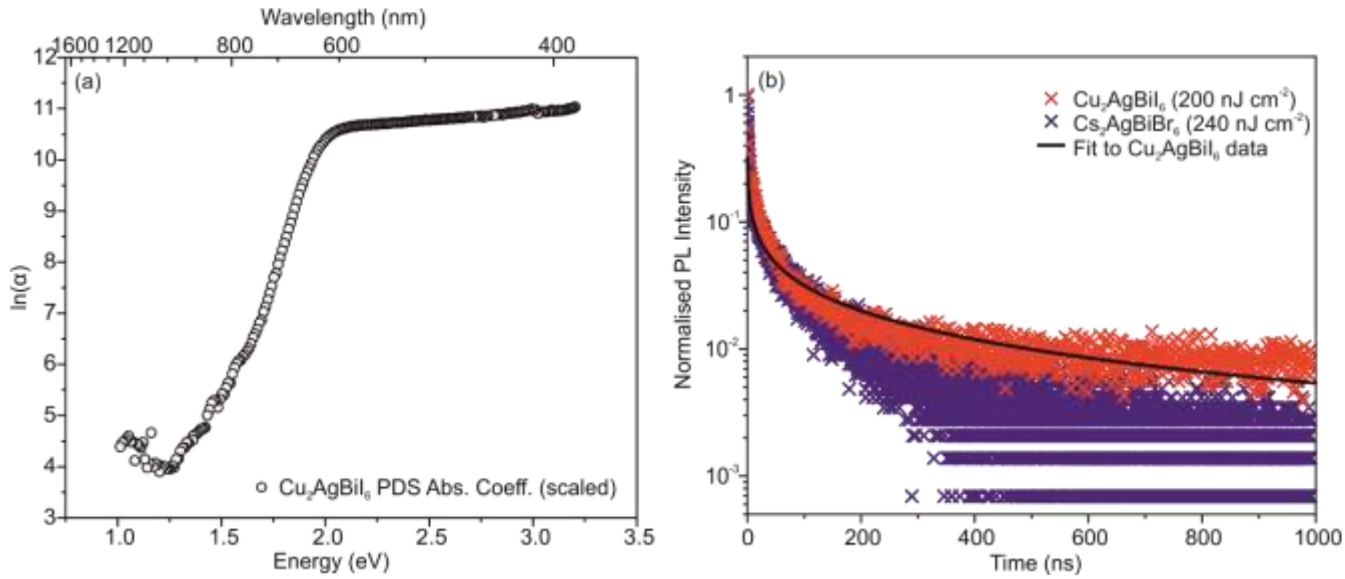

Figure S16. (a) The natural logarithm of the absorption coefficient measured by Photothermal Deflection Spectroscopy(PDS), after it was scaled to match the FTIR absorption coefficient data (raw data and scaled data shown in Figures S12a and b, respectively), showing sub-band gap states down to 1.25 eV. (b) Transient photoluminescence measurement on Cu_2AgBiI_6 (red) fitted by a stretched exponential function (black) with an average lifetime of 33 ns, compared to that of $Cs_2AgBiBr_6$ (blue) with an average lifetime pf 10 ns, reproduced from Longo *et al*.[23] The Cu_2AgBiI_6 and $Cs_2AgBiBr_6$ films were measured at excitation fluences of 200 nJ cm^{-2} and 240 nJ cm^{-2}, respectively.

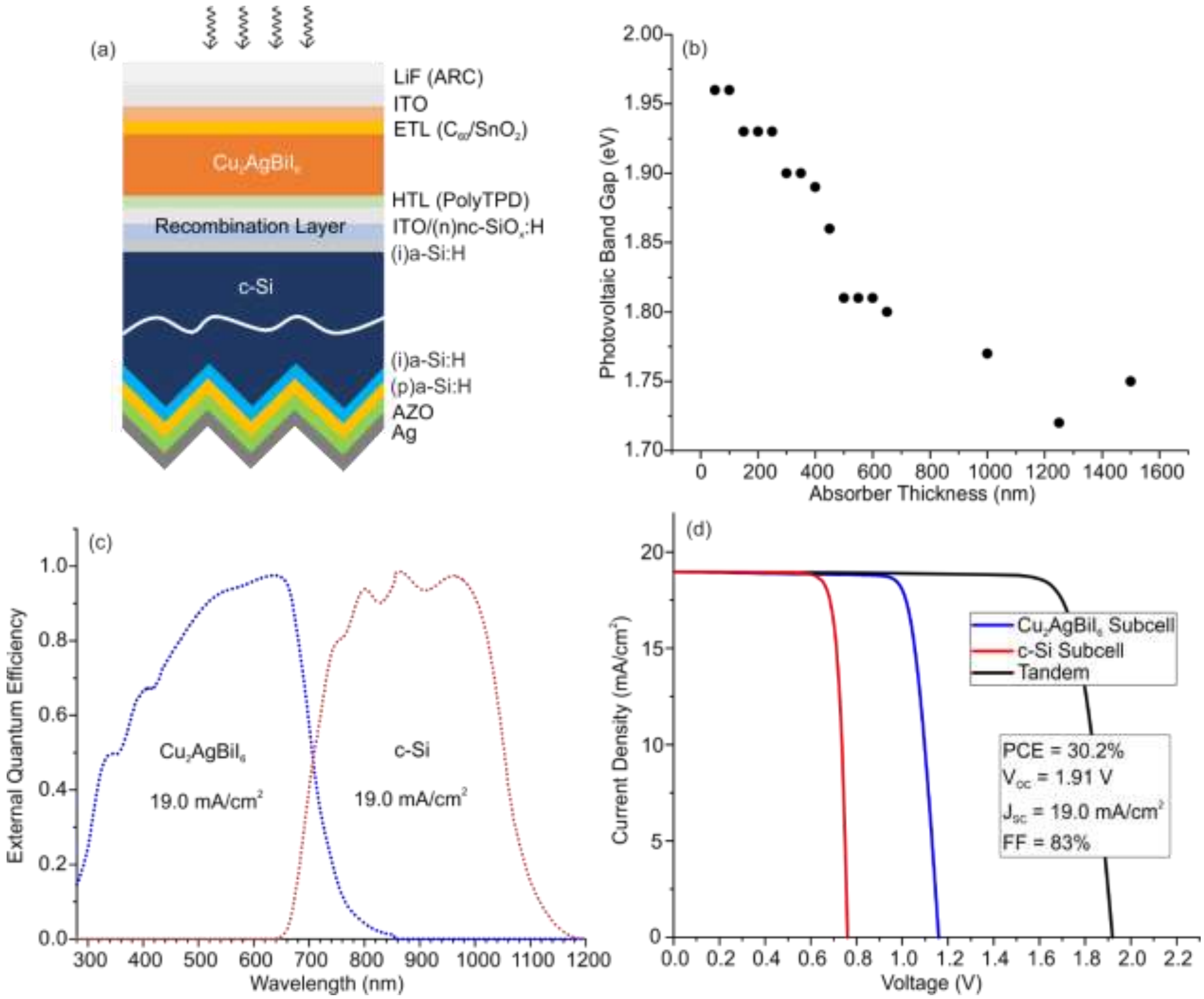

Figure S17. (a) The structure of the cell in the optical modelling LiF/ITO/SnO_2/C60/Cu_2AgBiI_6/PolyTPD/ITO/nc-SiO_x:H/(i)a-Si:H/c-Si/(i)a-Si:H/(p)a-Si:H/AZO/Ag. (b) The photovoltaic (PV) band gap, defined as the energy of the inflection point on the absorption edge, for Cu_2AgBiI_6 films of varying thicknesses. (c) The external quantum efficiency (EQE) and (d) the J-V curve of a simulated Cu_2AgBiI_6 on c-Si tandem solar cell, using a transfer matrix optical model, coupled with detailed balance. The diode parameters used were extracted from lead halide perovskite and c-Si J-V curves reported in literature (see SI for more details).

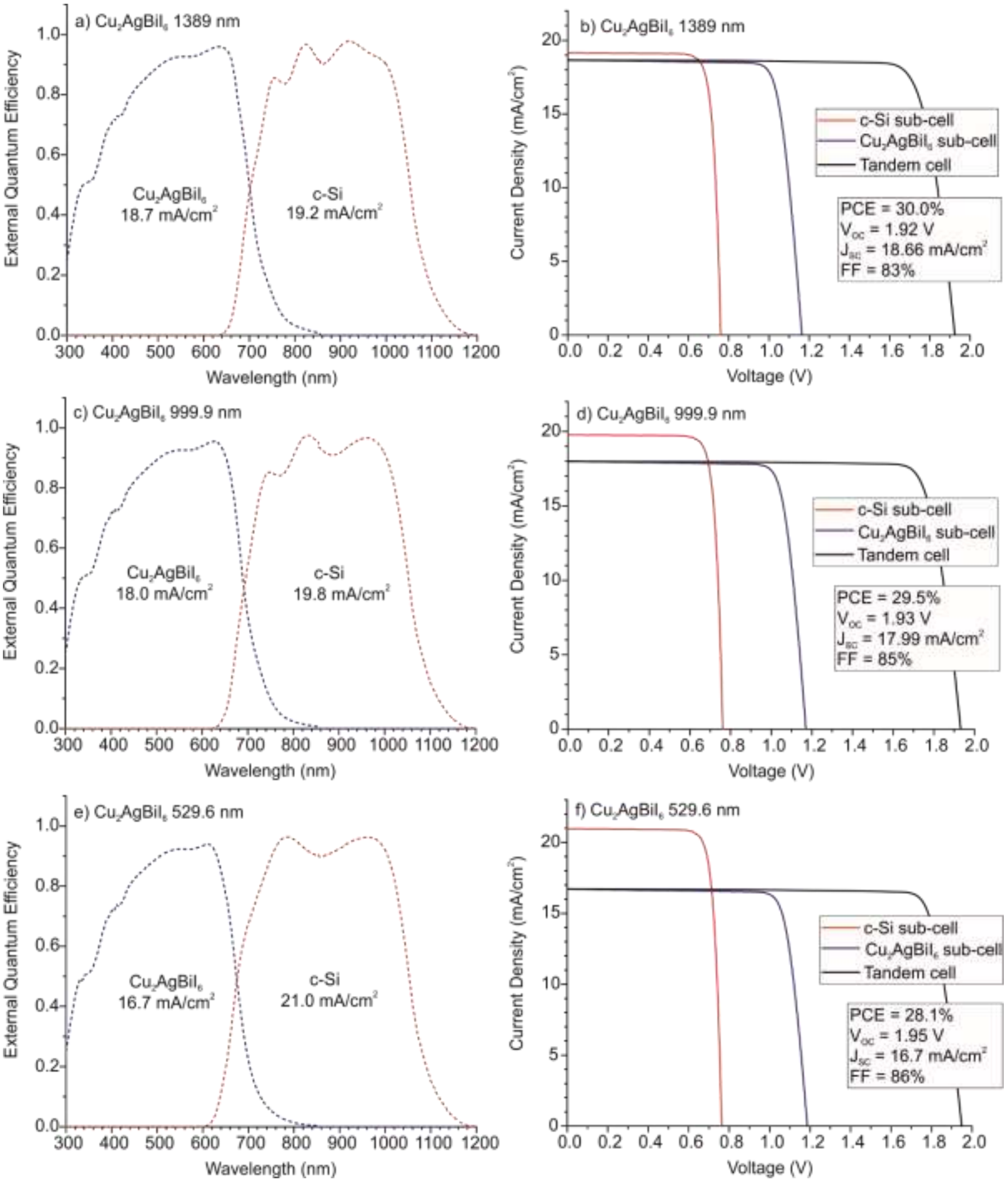

Figure S18. The external quantum efficiencies (EQE) (left) and the J-V curves (right) of simulated Cu_2AgBiI_6 films of varying thickness on c-Si tandem cells, using a transfer matrix optical model, coupled with detailed balance. The diode parameters used were extracted from lead halide perovskite and c-Si J-V curves reported in literature (see SI for more details). The search bounds for Cu_2AgBiI_6 film thicknesses were 1200–1400 nm, 800–1000 nm, and 400–530 nm for the films that optimised at thicknesses of 1389 nm, 999.9 nm and 529.6 nm, respectively. LiF film thickness optimised at 97.3 nm, 96.8 nm, 95.3 nm for the 1389 nm, 999.9 nm and 529.6 nm Cu_2AgBiI_6 film thicknesses, respectively.

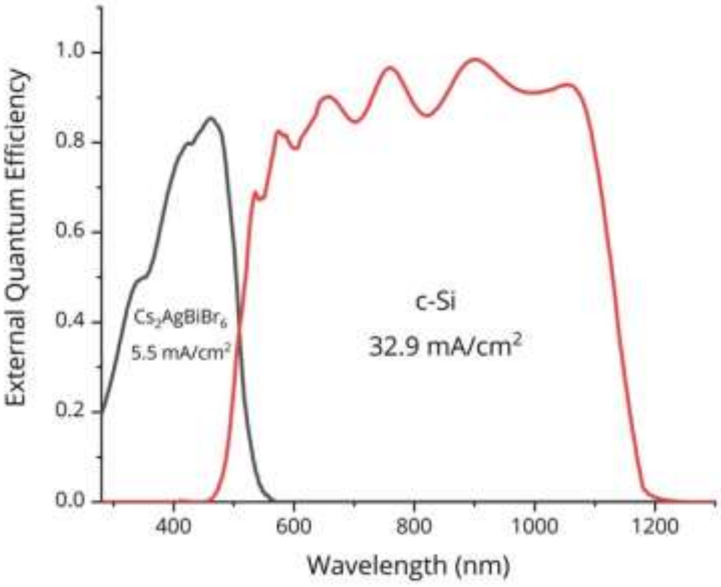

S19. The optical response of a $Cs_2AgBiBr_6$ on silicon tandem was modelled using the transfer matrix method.[13] The thickness of the $Cs_2AgBiBr_6$ (100 nm – 1000 nm) and the anti-reflective coating LiF (10nm – 150 nm) was varied using a differential evolution algorithm till the limiting tandem current was maximised. The optimised device structure was LiF (110 nm)/ITO (80 nm)/SnO2 (5 nm)/C60 (10 nm)/$Cs_2AgBiBr_6$ (989 nm)/PolyTPD (5 nm)/ITO (25 nm)/nc-SiO_x:H (110 nm)/i-a-Si:H (5 nm)/i-c-Si (250 um)/i-a-Si:H (5 nm)/p-a-Si:H (5 nm)/AZO (70 nm)/Ag (400 nm). The c-Si sub-cell (32.9 mA/cm^2) generates six times as much as current as the $Cs_2AgBiBr_6$ cell (5.5 mA/cm^2). This huge current mismatch makes $Cs_2AgBiBr_6$ impractical as the top cell of a Si based tandem.

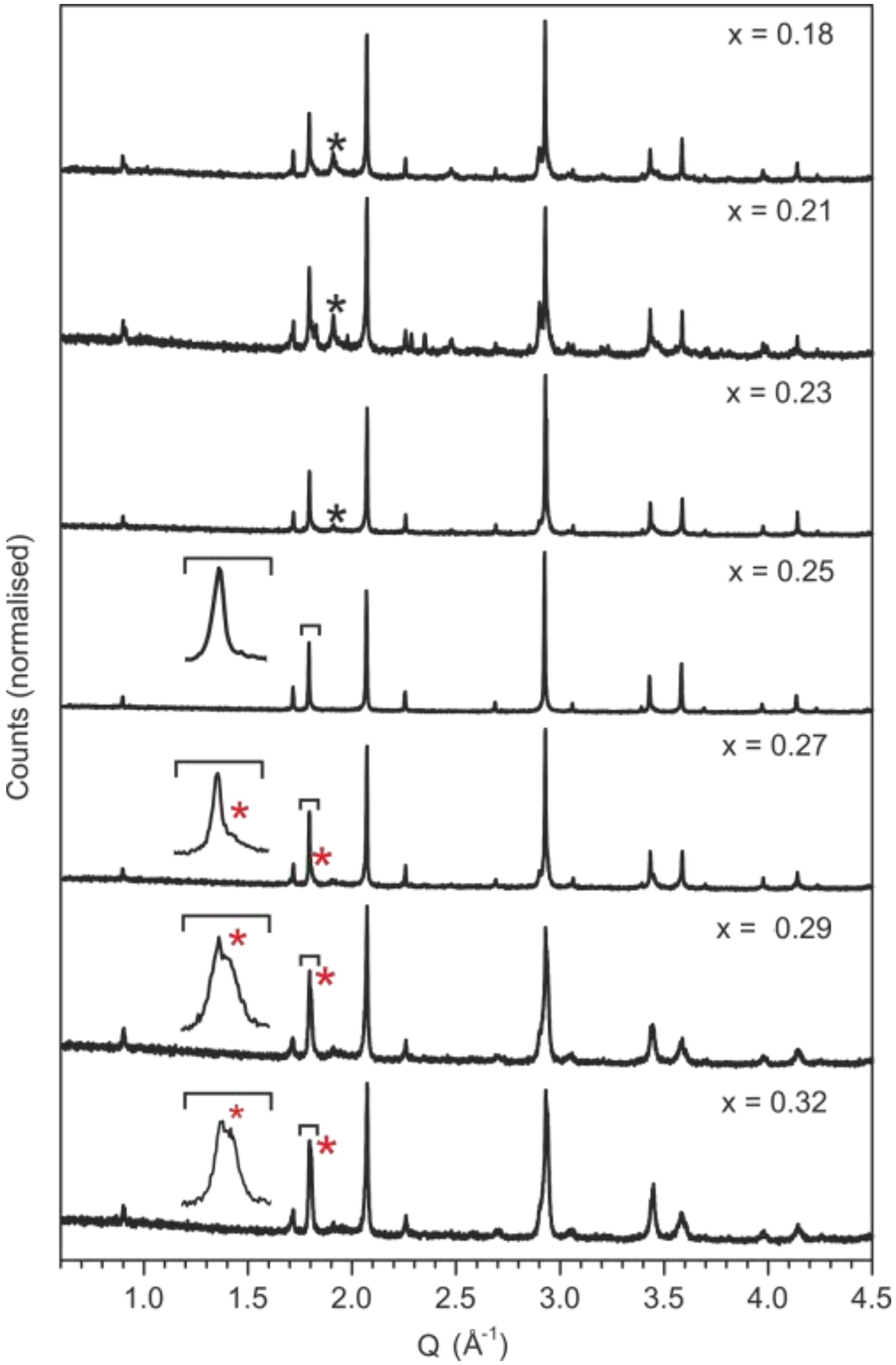

Figure S20. PXRD patterns for the compositional screening of $Cu_{3x}Bi_{1-x}I_3$ carried out between $0.18 \leq x \leq 0.32$. A pure $CuBiI_4$ powder was obtained for the nominal composition corresponding to $x = 0.25$. For values of $x < 0.25$ BiI_3 impurities were seen (black asterisks). For values of $x > 0.25$ small amounts of CuI impurities were seen which appear on the shoulders of the $CuBiI_4$ peaks (red asterisks). The expanded view of the peak at 1.8 Å^{-1} in the PXRD patterns of $x > 0.25$, shows the amount of CuI increases with increasing x above 0.25.

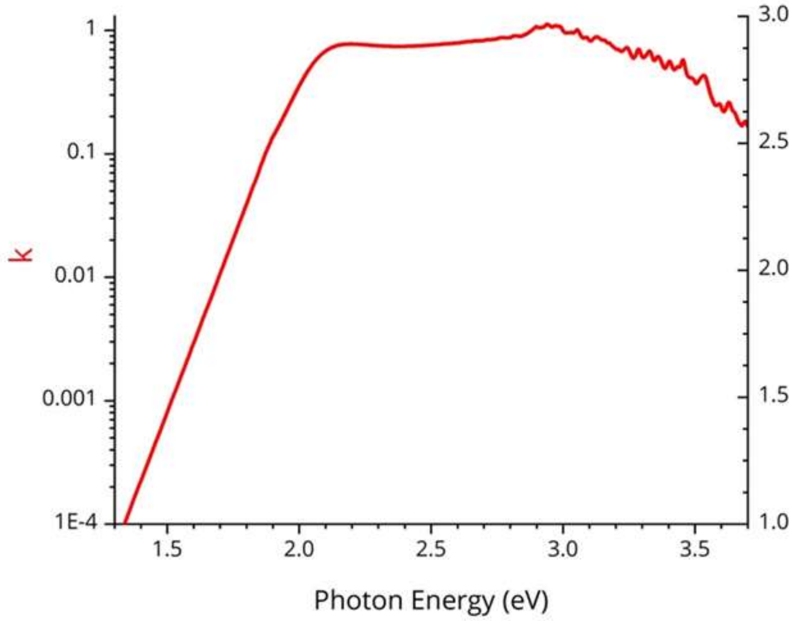

Figure S21. The "synthetic" extinction co-efficient for Cu_2AgBiI_6 which was used as input for the optical calculations, as described in the optical modelling .

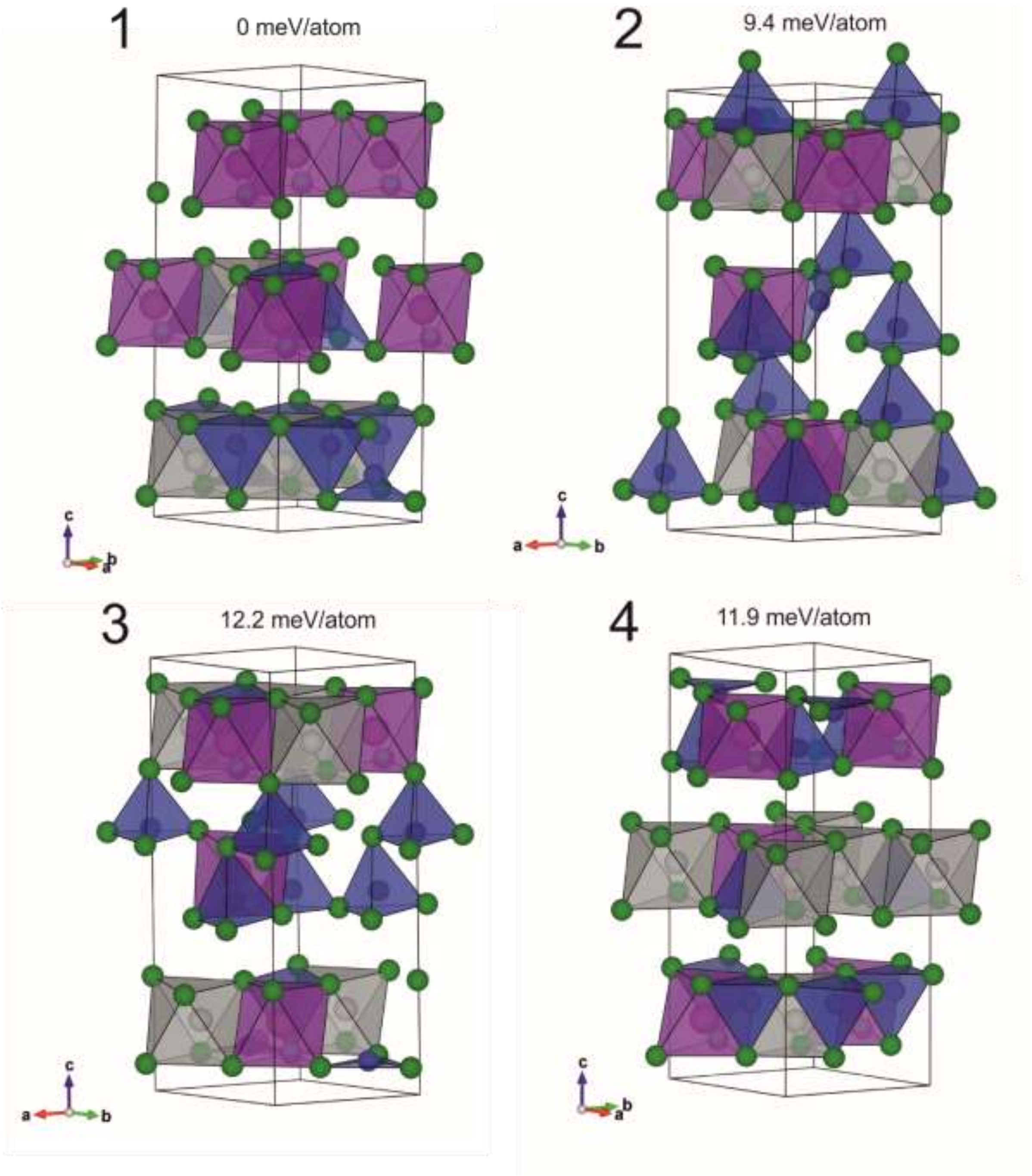

Figure S22. The optimised geometry of the lowest energy configurations arising from each of the four independent ChemDASH calculations computed using the van der Waals functional, optB86b-vdW.[20] The computed energy for each configuration is given relative to configuration 1, the lowest energy overall. The green, blue, grey and pink spheres/polyhedra represent I^-, Cu^+, Ag^+ and Bi^{3+} ions respectively. These low energy configurations represent different ordered arrangements of the cations consistent with the average structure determined experimentally.

Table S1. The twinning matrix and contribution of each twin indexing the Cu_2AgBiI_6 100K SCXRD data with a 4-fold twin of smaller trigonal unit cells.

	Twin 1
Refined mass fraction (%)	24.9(1.2)
Isolated reflections	728
Overlapped reflections	733
Twin matrix	$\begin{bmatrix} 1 & 0 & 0 \\ 0 & 1 & 0 \\ 0 & 0 & 1 \end{bmatrix}$

	Twin 2
Refined mass fraction (%)	25.2(7)
Isolated reflections	729
Overlapped reflections	733
Twin matrix	$\begin{bmatrix} -0.3335 & -0.6669 & -0.1666 \\ 1.0003 & 1.0005 & -0.0001 \\ 2.6652 & -2.6675 & 0.3338 \end{bmatrix}$

	Twin 3
Refined mass fraction (%)	25.0(7)
Isolated reflections	729
Overlapped reflections	733
Twin matrix	$\begin{bmatrix} -0.6662 & -0.3332 & 0.1667 \\ -0.3340 & -0.6671 & -0.1667 \\ 2.6660 & -2.6665 & 0.3331 \end{bmatrix}$

	Twin 4
Refined mass fraction (%)	24.9(7)
Isolated reflections	745
Overlapped reflections	733
Twin matrix	$\begin{bmatrix} 0.9982 & 0.9973 & -0.0001 \\ -0.6647 & -0.3313 & 0.1663 \\ 2.6594 & -2.6646 & 0.3327 \end{bmatrix}$

Table S2. Cu_2AgBiI_6 structural refinement of single crystal X-ray data collected at 100 K.

Identification code	Cu2AgBiI6_SCXRD_100K
Empirical formula	$Cu_{2.15}Ag_{1.04}Bi_{0.92}I_{6.00}$
Formula weight	1202.46
Temperature (K)	100(2)
Crystal system	trigonal
Space group	$R\overline{3}m$
a (Å)	4.2749(3)
c (Å)	20.9395(16)
Volume (Å^3)	331.40(5)
Z	1.19988
ρ_{calc} (g/cm^3)	6.025
μ (mm^{-1})	31.009
F(000)	506.0
Crystal size (mm)	0.02 × 0.03 × 0.01
Radiation	MoKα (λ = 0.71073)
2θ range for data collection (°)	5.836 to 55.51
Index ranges	-5 ≤ h ≤ 5, -5 ≤ k ≤ 5, -26 ≤ l ≤ 27
Reflections collected	501
Independent reflections	501 [R_{sigma} = 0.0204]
Data/restraints/parameters	501/0/12
Goodness-of-fit on F^2	1.130
Final R indexes [I≥2σ]	R_1 = 0.0441, wR_2 = 0.1024
Final R indexes [all data]	R_1 = 0.0495, wR_2 = 0.1053
Largest diff. peak/hole (eÅ^{-3})	2.95/-4.31

Table S3. Bond distances and bond angles for the Cu_2AgBiI_6 structure refined against 100K SCXRD data.

**Cu_2AgBiI_6 100K SCXRD**						
***i*-$CdCl_2$**						
**Bond distances (Å)**			**Bond angles (°)**			
Oct1	I1	3.0471(9)	I1	Oct1	I1	89.09(4)
Cu1	I1	2.81(4)	I1	Oct1	I1	90.91(4)
Cu1	I1	2.583(12)	I1	Cu1	I1	107.1(9)
Cu2	I1	2.73(4)	I1	Cu1	I1	111.7(8)
Cu2	I1	2.558(11)	I1	Cu2	I1	105.2(9)
I1	I1	4.206(3)	I1	Cu2	I1	113.3(8)
I1	I1	4.2749(3)	I1	I1	I1	58.96(4)
I1	I1	4.343(3)	I1	I1	I1	59.46(2)
			I1	I1	I1	60.519(19)
			I1	I1	I1	60.000(5)

Table S4. Relative errors of the 1σ standard deviation in the measured compositions obtained by SEM and TEM EDX. CuI and AgI were purchased, the remaining were synthesised.

**Sample, measurement technique**	**Relative 1σ error in measured composition (%)**			
	**Cu**	**Ag**	**Bi**	**I**
**CuI (SEM EDX)**	3			2
**AgI (SEM EDX)**		2		2
**BiI_3 (SEM EDX)**			4	2
**$CuBiI_4$ (SEM EDX)**	4		6	2
**CuI (TEM EDX)**	3			3
**AgI (TEM EDX)**		2		2
**BiI_3 (TEM EDX)**			5	2
**Cu_2AgBiI_6 powder (TEM EDX)**	7	5	8	2
**Cu_2AgBiI_6 film (TEM EDX)**	4	7	13	3

Table S5. The stack used as input for the Transfer Matrix Calculations

LiF	110 nm
ITO[24]	80 nm
SnO_2[25]	5 nm
C60[26]	10 nm
Cu_2AgBiI_6	1710 nm
PolyTPD[27]	5 nm
ITO[24]	25 nm
Nc-SiO_x:H[24]	110 nm
(i)a-Si:H[28]	5 nm
c-Si[29]	250 µm
(i)a-Si:H[28]	5 nm
(p)a-Si:H[28]	5 nm
AZO[30]	70 nm
Ag[31]	400 nm

References

1. Sansom, H. C.; Whitehead, G. F. S.; Dyer, M. S.; Zanella, M.; Manning, T. D.; Pitcher, M. J.; Whittles, T. J.; Dhanak, V. R.; Alaria, J.; Claridge, J. B.; Rosseinsky, M. J., $AgBiI_4$ as a Lead-Free Solar Absorber with Potential Application in Photovoltaics. *Chem. Mater.* **2017,** *29* (4), 1538-1549.
2. Degen, T.; Sadki, M.; Bron, E.; König, U.; Nénert, G., The HighScore Suite. *Powder Diffr.* **2014,** *29* (S2), S13-S18.
3. Momma, K.; Izumi, F., VESTA: a Three-Dimensional Visualization System for Electronic and Structural Analysis. *J. Appl. Crystallogr.* **2008,** *41* (3), 653-658.
4. Sheldrick, G. M., A Short History of SHELX. *Acta Crystallogr. A* **2008,** *64*, 112-122.
5. Dolomanov, O. V.; Bourhis, L. J.; Gildea, R. J.; Howard, J. A. K.; Puschmann, H., OLEX2: a Complete Structure Solution, Refinement and Analysis Program. *J. Appl. Crystallogr.* **2009,** *42* (2), 339-341.
6. Parrott, E. S.; Green, T.; Milot, R. L.; Johnston, M. B.; Snaith, H. J.; Herz, L. M., Interplay of Structural and Optoelectronic Properties in Formamidinium Mixed Tin–Lead Triiodide Perovskites. *Adv. Funct. Mater.* **2018,** *28* (33), 1802803.
7. Lindsey, C. P.; Patterson, G. D., Detailed Comparison of the Williams–Watts and Cole–Davidson Functions. *J. Chem. Phys.* **1980,** *73* (7), 3348-3357.
8. Wright, A. D.; Verdi, C.; Milot, R. L.; Eperon, G. E.; Pérez-Osorio, M. A.; Snaith, H. J.; Giustino, F.; Johnston, M. B.; Herz, L. M., Electron–Phonon Coupling in Hybrid Lead Halide Perovskites. *Nat. Commun.* **2016,** *7* (1), 11755.
9. Davies, C. L.; Filip, M. R.; Patel, J. B.; Crothers, T. W.; Verdi, C.; Wright, A. D.; Milot, R. L.; Giustino, F.; Johnston, M. B.; Herz, L. M., Bimolecular Recombination in Methylammonium Lead Triiodide Perovskite is an Inverse Absorption Process. *Nat. Commun.* **2018,** *9* (1), 293.
10. Wehrenfennig, C.; Eperon, G. E.; Johnston, M. B.; Snaith, H. J.; Herz, L. M., High Charge Carrier Mobilities and Lifetimes in Organolead Trihalide Perovskites. *Adv. Mater.* **2014,** *26* (10), 1584-1589.
11. Elliott, R. J., Intensity of Optical Absorption by Excitons. *Phys. Rev.* **1957,** *108* (6), 1384-1389.
12. Sell, D. D.; Lawaetz, P., New Analysis of Direct Exciton Transitions: Application to GaP. *Phys. Rev. Lett.* **1971,** *26* (6), 311-314.
13. Katsidis, C. C.; Siapkas, D. I., General Transfer-Matrix Method for Optical Multilayer Systems with Coherent, Partially Coherent, and Incoherent Interference. *Appl. Opt.* **2002,** *41* (19), 3978-3987.
14. Saliba, M.; Matsui, T.; Domanski, K.; Seo, J.-Y.; Ummadisingu, A.; Zakeeruddin, S. M.; Correa-Baena, J.-P.; Tress, W. R.; Abate, A.; Hagfeldt, A.; Grätzel, M., Incorporation of Rubidium Cations into Perovskite Solar Cells Improves Photovoltaic Performance. *Science* **2016,** *354* (6309), 206-209.
15. Masuko, K.; Shigematsu, M.; Hashiguchi, T.; Fujishima, D.; Kai, M.; Yoshimura, N.; Yamaguchi, T.; Ichihashi, Y.; Mishima, T.; Matsubara, N.; Yamanishi, T.; Takahama, T.; Taguchi, M.; Maruyama, E.; Okamoto, S., Achievement of More Than 25% Conversion Efficiency With Crystalline Silicon Heterojunction Solar Cell. *IEEE J PHOTOVOLT* **2014,** *4* (6), 1433-1435.
16. Ohta, K.; Ishida, H., Comparison Among Several Numerical Integration Methods for Kramers-Kronig Transformation. *Appl. Spectrosc.* **1988,** *42* (6), 952-957.
17. Kresse, G.; Furthmüller, J., Efficient Iterative Schemes for Ab Initio Total-Energy Calculations using a Plane-Wave Basis Set. *Phys. Rev. B* **1996,** *54* (16), 11169-11186.
18. Kresse, G.; Joubert, D., From Ultrasoft Pseudopotentials to the Projector Augmented-Wave Method. *Phys. Rev. B* **1999,** *59* (3), 1758-1775.
19. Sharp, P. M.; Dyer, M. S.; Darling, G. R.; Claridge, J. B.; Rosseinsky, M. J., Chemically Directed Structure Evolution for Crystal Structure Prediction. *Phys. Chem. Chem. Phys.* **2020,** *22* (32), 18205-18218.
20. Klimeš, J.; Bowler, D. R.; Michaelides, A., Van der Waals Density Functionals Applied to Solids. *Phys. Rev. B* **2011,** *83* (19), 195131.
21. Sun, J.; Ruzsinszky, A.; Perdew, J. P., Strongly Constrained and Appropriately Normed Semilocal Density Functional. *Phys. Rev. Lett.* **2015,** *115* (3), 036402.

22. Fourcroy, P. H.; Carre, D.; Thevet, F.; Rivet, J., Structure du Tetraiodure de Cuivre(I) et de Bismuth(III), $CuBiI_4$. *Acta Crystallogr. C* **1991,** *47* (10), 2023-2025.
23. Longo, G.; Mahesh, S.; Buizza, L. R. V.; Wright, A. D.; Ramadan, A. J.; Abdi-Jalebi, M.; Nayak, P. K.; Herz, L. M.; Snaith, H. J., Understanding the Performance-Limiting Factors of $Cs_2AgBiBr_6$ Double-Perovskite Solar Cells. *ACS Energy Lett.* **2020**, 2200-2207.
24. Mazzarella, L.; Lin, Y.-H.; Kirner, S.; Morales-Vilches, A. B.; Korte, L.; Albrecht, S.; Crossland, E.; Stannowski, B.; Case, C.; Snaith, H. J.; Schlatmann, R., Infrared Light Management Using a Nanocrystalline Silicon Oxide Interlayer in Monolithic Perovskite/Silicon Heterojunction Tandem Solar Cells with Efficiency above 25%. *Adv. Energy Mater.* **2019,** *9* (14), 1803241.
25. Pan, S. S.; Zhang, Y. X.; Teng, X. M.; Li, G. H.; Li, L., Optical Properties of Nitrogen-Doped SnO2 Films: Effect of the Electronegativity on Refractive Index and Band Gap. *J. Appl. Phys.* **2008,** *103* (9), 093103.
26. Ren, S. L.; Wang, Y.; Rao, A. M.; McRae, E.; Holden, J. M.; Hager, T.; Wang, K.; Lee, W. T.; Ni, H. F.; Selegue, J.; Eklund, P. C., Ellipsometric Determination of the Optical Constants of C60 (Buckminsterfullerene) Films. *Appl. Phys. Lett.* **1991,** *59* (21), 2678-2680.
27. Shibata, M.; Sakai, Y.; Yokoyama, D., Advantages and Disadvantages of Vacuum-Deposited and Spin-Coated Amorphous Organic Semiconductor Films for Organic Light-Emitting Diodes. *J. Mater. Chem. C* **2015,** *3* (42), 11178-11191.
28. Holman, Z. C.; Filipič, M.; Descoeudres, A.; De Wolf, S.; Smole, F.; Topič, M.; Ballif, C., Infrared Light Management in High-Efficiency Silicon Heterojunction and Rear-Passivated Solar Cells. *J. Appl. Phys.* **2013,** *113* (1), 013107.
29. Green, M. A., Self-Consistent Optical Parameters of Intrinsic Silicon at 300K including Temperature Coefficients. *Sol. Energy Mater. Sol. Cells* **2008,** *92* (11), 1305-1310.
30. Treharne, R. E.; Seymour-Pierce, A.; Durose, K.; Hutchings, K.; Roncallo, S.; Lane, D., Optical Design and Fabrication of Fully Sputtered CdTe/CdS Solar Cells. *J. Phys. Conf. Ser.* **2011,** *286*, 012038.
31. Ciesielski, A.; Skowronski, L.; Trzcinski, M.; Szoplik, T., Controlling the Optical Parameters of Self-Assembled Silver Films with Wetting Layers and Annealing. *Appl. Surf. Sci.* **2017,** *421*, 349-356.
